# Infectious Disease Modeling of Social Contagion in Networks

**DOI:** 10.1371/journal.pcbi.1000968

**Published:** 2010-11-04

**Authors:** Alison L. Hill, David G. Rand, Martin A. Nowak, Nicholas A. Christakis

**Affiliations:** 1 Program for Evolutionary Dynamics, Harvard University, Cambridge, Massachusetts, United States of America; 2 Biophysics Program and Harvard-MIT Division of Health Sciences and Technology, Harvard University, Cambridge, Massachusetts, United States of America; 3 Department of Psychology and Berkman Center for Internet and Society, Harvard University, Cambridge, Massachusetts, United States of America; 4 Department of Mathematics, Harvard University, Cambridge, Massachusetts, United States of America; 5 Department of Organismic and Evolutionary Biology, Harvard University, Cambridge, Massachusetts, United States of America; 6 Department of Medicine, Harvard Medical School, Boston, Massachusetts, United States of America; 7 Department of Health Care Policy, Harvard Medical School, Boston, Massachusetts, United States of America; 8 Department of Sociology, Harvard University, Cambridge, Massachusetts, United States of America; University of Washington, United States of America

## Abstract

Many behavioral phenomena have been found to spread interpersonally through social networks, in a manner similar to infectious diseases. An important difference between social contagion and traditional infectious diseases, however, is that behavioral phenomena can be acquired by non-social mechanisms as well as through social transmission. We introduce a novel theoretical framework for studying these phenomena (the SISa model) by adapting a classic disease model to include the possibility for ‘automatic’ (or ‘spontaneous’) non-social infection. We provide an example of the use of this framework by examining the spread of obesity in the Framingham Heart Study Network. The interaction assumptions of the model are validated using longitudinal network transmission data. We find that the current rate of becoming obese is 2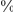 per year and increases by 0.5 percentage points for each obese social contact. The rate of recovering from obesity is 4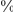 per year, and does not depend on the number of non-obese contacts. The model predicts a long-term obesity prevalence of approximately 42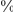, and can be used to evaluate the effect of different interventions on steady-state obesity. Model predictions quantitatively reproduce the actual historical time course for the prevalence of obesity. We find that since the 1970s, the rate of recovery from obesity has remained relatively constant, while the rates of both spontaneous infection and transmission have steadily increased over time. This suggests that the obesity epidemic may be driven by increasing rates of becoming obese, both spontaneously and transmissively, rather than by decreasing rates of losing weight. A key feature of the SISa model is its ability to characterize the relative importance of social transmission by quantitatively comparing rates of spontaneous versus contagious infection. It provides a theoretical framework for studying the interpersonal spread of any state that may also arise spontaneously, such as emotions, behaviors, health states, ideas or diseases with reservoirs.

## Author Summary

Information, trends, behaviors and even health states may spread between contacts in a social network, similar to disease transmission. However, a major difference is that as well as being spread infectiously, it is possible to acquire this state spontaneously. For example, you can gain knowledge of a particular piece of information either by being told about it, or by discovering it yourself. In this paper we introduce a mathematical modeling framework that allows us to compare the dynamics of these social contagions to traditional infectious diseases. We can also extract and compare the rates of spontaneous versus contagious acquisition of a behavior from longitudinal data and can use this to predict the implications for future prevalence and control strategies. As an example, we study the spread of obesity, and find that the current rate of becoming obese is about 2 per year and increases by 0.5 percentage points for each obese social contact, while the rate of recovering from obesity is 4 per year. The rates of spontaneous infection and transmission have steadily increased over time since 1970, driving the increase in obesity prevalence. Our model thus provides a quantitative way to analyze the strength and implications of social contagions.

## Introduction

Social network effects are of great importance for understanding human behavior. People interact with a varying number of individuals and with some individuals more than others, and this affects behavior in fundamental ways. Sociologists have long studied social influence through networks, and networks now routinely appear in investigations from other fields, including economics [1], physics [2], public health [3] and scientific publishing [4], [5]. Extensive reviews of social networks analysis, including investigations of their structure and their effect on social dynamics, include Mitchell [6], Wasserman [7],Watts [2], Rogers [8], Jackson [1], and Smith [9]. Networks have also long been known to be important in many areas of biology (reviewed by [10]), including ecological food webs and the evolution of cooperation [11]–[14]. Social networks have also been studied as determinants of health (reviewed by Smith [9]), ranging from determining the patterns of infectious disease spread [15] to the propagation of phenomena such as emotions [16]–[18], smoking cessation [19], obesity [20], suicide [21], altruism [22], anti-social behavior [23], and online health forum participation [24]. These studies suggest that on top of the physical environment, the social environment can also be an important contributor to health. They have lead to suggestions that public health interventions must be designed that work with the network structure and that the network can be exploited to spread health related information [9], [25].

Within network studies, much work has focused on how information, trends, behaviors and other entities spread between the individuals in social networks. These processes are generally referred to as ‘contagion’. Such suggestions of contagious dynamics and the possible relevance of network structure can be rigorously examined using mathematical models of contagious processes. These can then be used to obtain accurate measures of expected prevalences, interventional efficacy, and optimized information flow. Many previous models have been proposed to study influential interactions between individuals. Most of these have considered well-mixed populations, although more recent work has focused on network-structured populations. The most well studied are classic epidemiological models (like SIS and SIR) for the spread of microbial infectious diseases [26], including spread in network-structured populations [27]–[30], [31], [15]. Various related processes have been used to model social influence, with important contributions including the same epidemiological models [32], [33], diffusion models [8], [34]–[38], statistical mechanics type interactions [39], [40], and threshold models [41](reviewed by Jackson [1] and Newman et al. [42]).

Each of these models, however, has one or more properties that are problematic for studying social contagion. Many do not capture the probabilistic nature of contagion, or the asymmetry inherent in traditional infectious disease (where the infected state spreads through social contagion whereas the non-infected state does not). Others only consider well-mixed populations, where everyone is influenced by everyone else, ignoring the effect of network structure. Most models inspired by epidemiology are not directly applicable to the social spread of other phenomenon, because many phenomena that spread by social contagion may also arise spontaneously. That is, it is possible to adopt a trend or behavior, or obtain information, from an outside source, without directly ‘catching’ it from a contact in the network. In other words, on top of the probability of obtaining the infection from each infected contact, there is also a non-zero probability of ‘automatically’ obtaining the infection, independent of the local network. This ‘automatic’ non-social infection is not included in traditional infectious disease models. Economic models for the diffusion of innovations, based on early work by Bass [34], do take into account ‘automatic’ infection. Individuals move from ‘susceptible’ (non-adopter) to an infected (adopter) state by adopting a new product or idea, influenced by both social and non-social factors. However, these models do not allow for recovery; because the innovation adoptions are assumed to be permanent changes in behavior, individuals never move back to a susceptible state. This results in the entire population becoming adopters at equilibrium. This does not reflect the dynamics of many phenomena that spread socially, which may be repeatedly acquired and lost (for example, happiness or obesity). Through a balance of infection and recovery, a steady-state with multiple states of individuals coexisting can be reached. Finally, most previous models make assumptions about the type of interaction between individuals, the particulars of which are not usually validated with real data. Yet, long term behavior of a model and the prevention strategies it suggests can depend critically on the specifics of the interaction assumptions.

Here, we introduce a new model to study the spread of entities in a social network which has all of the important properties listed above. We then analyze its characteristics and show how it can be applied in different contexts. This model is an extension of the classical infectious disease model, combining features from other models mentioned above. It describes infections that can be contracted both spontaneously and through social (network-structured) transmission, and allows for recovery from infection. As an example, we focus on the spread of obesity in the Framingham Heart Study (FHS) network. The interaction assumptions of the model will be validated using longitudinal network transmission data. We show how we can quantitatively assess the values for the rate of adopting a trend spontaneously versus by contagion to determine the extent to which social transmission is important. We use it to predict prevalences and intervention effectiveness (i.e. get quantitative output, not just qualitative behavior). The results of this model are very different from models with other interaction assumptions, such as the ‘majority rules’ models. We will show that transmissive components are often small compared to the automatic component, but may still contribute materially to prevalence levels. Lastly, we will use pair-wise approximations to generate analytic results for infections in network-structured populations, as well as presenting simulations using a real social network.

## Methods

### Classic infectious disease modeling

 In the simplest infectious disease models [26], individuals are classified as occupying one of two states: ‘susceptible’, meaning they do not have the disease, and ‘infected’, meaning they do have the disease. The disease can be transmitted to a susceptible person when they come into contact with an infected person. The rate of this disease transmission from infected to susceptible is defined as 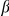, the *transmission rate*. Once an individual is infected, they recover from the disease at a constant rate 
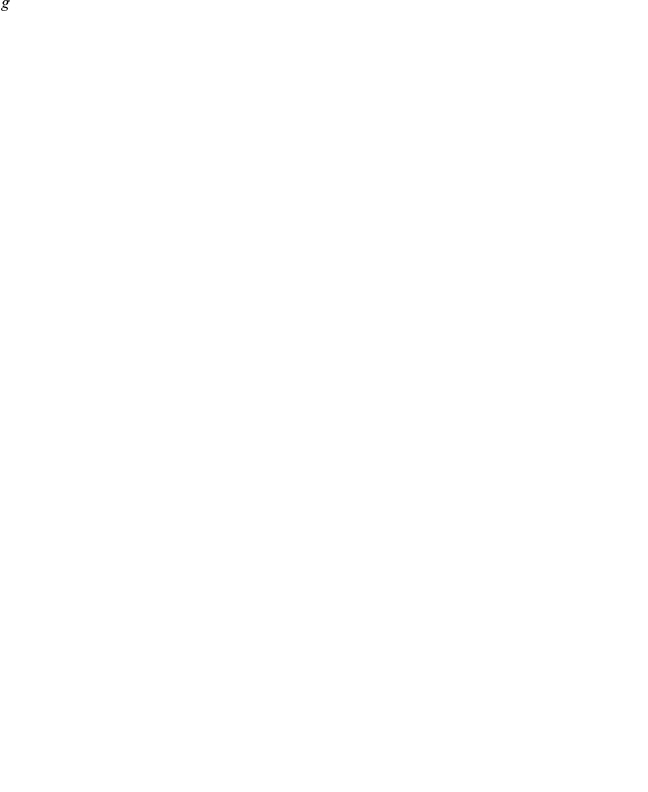
, regardless of their contacts with susceptibles or infecteds. In one class of disease models (susceptible-infected-recovered, or SIR), recovered individuals become immune to further infection and enter a ‘recovered’ state. However, behaviors, trends, health states, etc, can occur many times over an individual's life, and therefore we assume infected individuals return to the susceptible state after recovering. This form of susceptible-infected-susceptible (SIS) model is used to model infectious diseases that do not confer immunity, like many STDs.

### Application to social contagion

**Figure 1:**
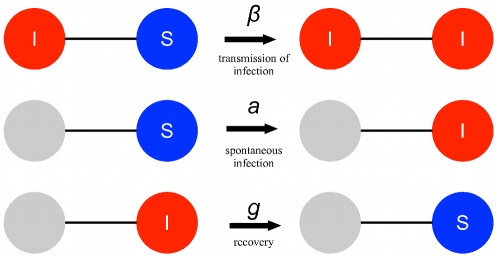
The SISa model of infection. There are three processes by which an individual's state can change. (i) An infected individual transmits infection to a susceptible contact with rate 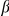. (ii) A susceptible individual spontaneously becomes infected at rate 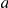, regardless of the state of their contacts. (iii) An infected individual returns to being susceptible at rate 
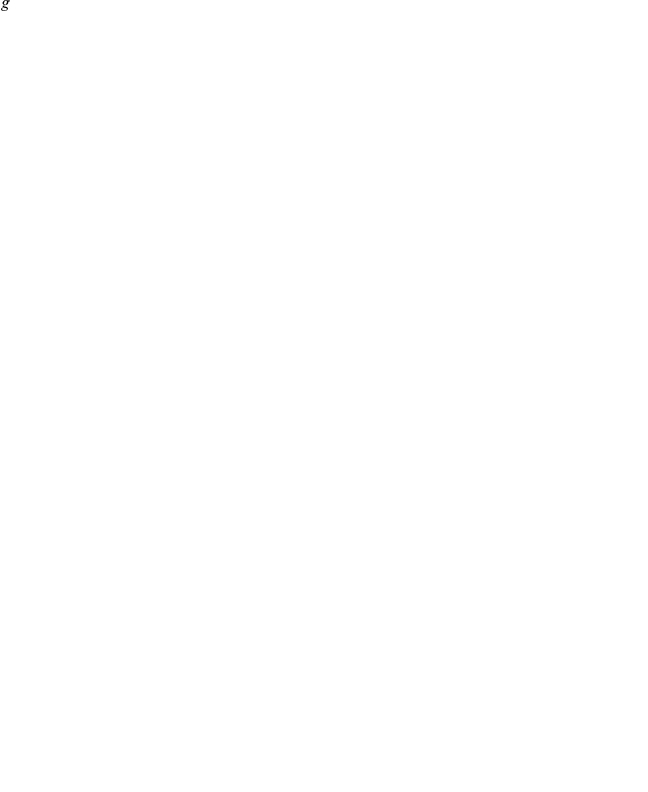
, independent of the state of their contacts.

In the standard SIS model, infection can only be transmitted by having a contact between an infected and a susceptible individual. Social ‘infections’, however, can also arise due to spontaneous factors other than transmission. Therefore, we extend the SIS model by adding a term whereby uninfected individuals spontaneously (or ‘automatically’) become infected at a constant rate 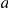, independent of infected contacts. A diagrammatic representation of our modified SIS model, which we will call SISa, is shown in Figure 1. The corresponding differential equations for a well-mixed population are described in Eq. 1
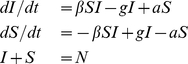
(1)where 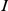 is the number of infected individuals, 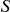 is the number of susceptible individuals, 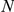 is the population size, 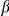 is the transmission rate, 
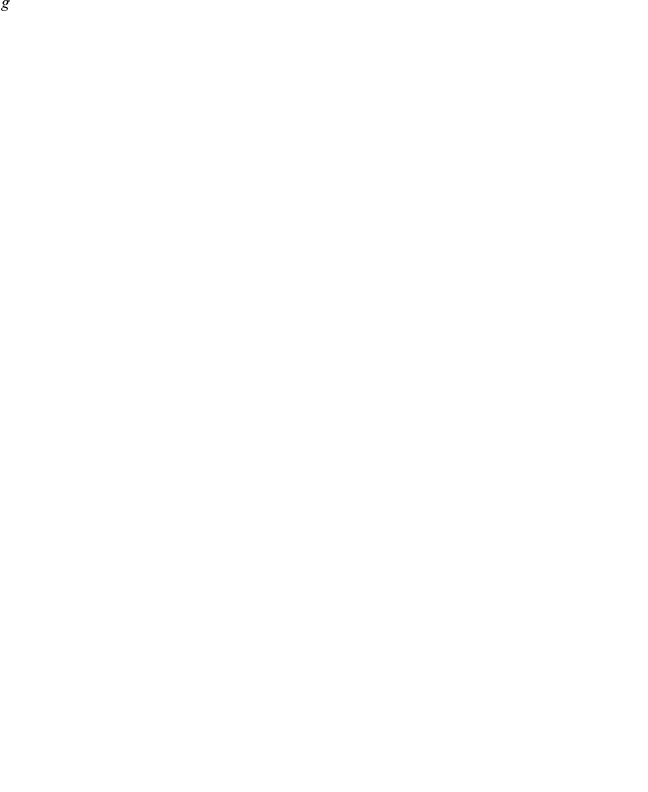
 is the recovery rate, and 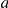 is the rate of spontaneous infection. This model assumes a constant population size and neglects birth and death. The SISa model is related to infectious disease models with ‘imports’ (migration of infecteds into the population), although here the rate of spontaneous infection is proportional to the number of susceptibles, while in import models it is a constant or proportional to the total population size.

In the infectious disease literature, a disease is said to be ‘endemic’ if a stable, non-zero fraction of the population is infected at steady state. If a single infected individual is introduced to a totally susceptible population, then the average number of secondary infections they cause before recovery is called the *basic reproductive ratio*, 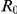. For the regular SIS model in a well-mixed population of N individuals, 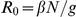. An epidemic, leading to an endemic equilibrium, only occurs for 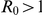, and hence 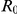 is a fundamental quantity used to describe and compare infectious diseases. For the SISa model, an epidemic occurs for all parameter values, due to the spontaneous infection term. Thus, social behaviors that can be adopted independently of neighbors mean that there is no longer a threshold for the behavior to become prevalent in a population, and even in the absence of contagion there would be a non-zero steady state prevalence. Because of this, there is not an obvious definition for 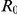 in the SISa model. The steady state fraction of infected individuals in a well-mixed population is given by Eq. 2.
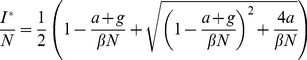
(2)


### Infectious diseases on networks

Traditional models of infection assume that the population is well-mixed. However, this assumption is unrealistic for many diseases, and also for the social spread of trends and behaviors. To account for the population structure, the infectious process can be constrained to take place on a social network. An infected individual can only pass their infection on to the suspectibles to whom they are connected. Properties of the infectious process thus depend on both the epidemiological parameters and the network structure, and there are often no longer simple analytic formulas to describe the reproductive ratio or steady state level of infection. For example, a property of disease spread on networks are *spatial correlations* (in the network sense) that arise between individuals in the same state. This correlation is defined as the ratio of the observed number of connections between two types of individuals to the number of connections expected if the positioning of individuals in the network was random. Spatial correlations of like individuals can be caused by an infective process spreading within a network [29], but may also be caused by confounding environmental factors which similarly influence the behavior of connected individuals, or the formation of contacts based on similar behavior (also called homophily). For a network of N individuals with a total of E connections between them, the correlation between two states X and Y is defined by:
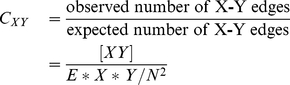
(3)


The correlation between infected individuals, 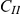, rises above one as the epidemic proceeds, due to cluster formation as infected individuals transmit to their contacts. Similarly, the correlation between infected and susceptible individuals, 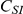, drops below one. The deviation of these correlations from 1 increases with (i) the ratio of transmissive infection (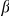) to spontaneous infection (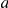) in our model (there are no correlations without a transmissive component), and (ii) the inter-connectivity (transitivity) of the network. As a result of these spatial correlations, diseases on networks can progress more slowly than their well-mixed counterparts, leading to lower basic reproductive ratios. However, heterogeneity in the number of contacts per individual acts to increase 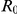. For two networks with the same average degree, if one has a larger variance in degree, then 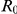 will be increased. Thus, it is possible for diseases on networks to have lower (or nonexistent) thresholds for endemic epidemics.

### Approximate pair-wise equations

There are no analytic methods to solve SIS-type dynamics on arbitrary networks without making approximations. Thus, simulations are a more accurate tool to explore theoretical disease dynamics in structured populations without making simplifying assumptions about the network structure. For scaled, well-mixed populations, the formulas given in the previous sections for 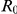 and 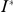 are good approximations if 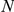 is replaced with 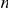, the average contacts at a given time, while fixed networks, especially if non-uniform and highly inter-connected, can deviate from these values significantly. We can use a pair-wise approximation [29], [43], [44] to formulate the infectious process on a network structure in terms of differential equations. The fundamental variables are numbers of individuals of each type, and also the pairs of individuals, [XY] (where the edges are not directional). Because [XY] = [YX], and the total individuals and total edges is constant, the system can be reduced to three equations.
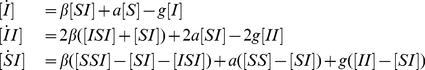
(4)


Here [XYZ] represents the number of situations where and X individual is connected to a Y individual who in turn is connected to a Z individual. We can approximate all these triples in terms of pairs, using a moment closure approximation ([43], Text S1), which then reduces the number of variables to three also. Then these equations can be simplified to
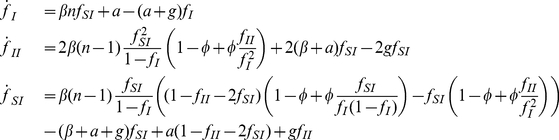
(5)with
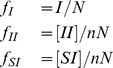
(6)where 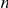 is the number of contacts each individual has and 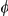 is the transitivity of the network (the ratio of triangles to triples). Having a simplified set of equations is very useful for understanding contagion dynamics in structured populations. Integrating equations is much faster than running simulations on large networks, and from them analytic results can be derived which allows determination of parameter dependence. These equations assume that the local neighborhood for each individual is identical, that is, everyone has the same number of contacts (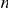) and the same 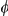. They thus take into account the effects of fixed network structure but not heterogeneities between individuals. In the Supplementary Information (Text S1) we have included the extension of these equations to include heterogeneities. These equations can be used to easily simulate disease spread and get expected steady state prevalences and correlations, which are very useful approximations and give insight into parameter dependence. Later, we will compare these equations to results from full simulations on realistic networks. When 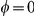 (which is approximately the case for most random graphs) we can get a closed-form solution for the prevalence at steady state:
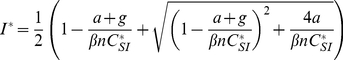
(7)

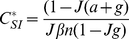
(8)

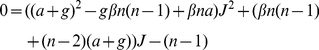
(9)


The result of a network structure is that the number of partnerships between susceptible and infected individuals quickly becomes less than if random, and so 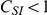. We can compare Eq. 7 to the well mixed result (Eq. 2), and see that the effect of the network is to lower the effective transmission rate by a factor of 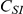, and hence lower the prevalence, due to these correlations that build up locally. The larger 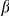 is compared to 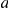, the more network effects are important. If infection is mostly automatic (when 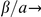
0), the network no longer matters. Equation 7 actually holds generally (for any homogeneous network and any 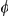 value), while Equations 8 and 9 are only applicable with 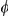 = 0.

Analyzing the n-regular pair-wise equations allows us to get analytic results and determine how and under what conditions network structure affects the spread of behaviors which are both spontaneously acquired and spread interpersonally. Although simple closed-form solutions do not exist when 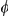 is non-zero, these equations can easily be integrated or numerically solved to get solutions. These equations ignore heterogeneities in the number of edges for different individuals, which can facilitate spread under some conditions (see supplement Text S1 for extension). Full stochastic simulations on large networks can be carried out to determine how and when the results differ.

## Results

### Calibrating model with FHS Network data

The SISa model provides a formal way for assessing the social contagion of trends and behaviors that may be repeatedly caught and recovered from. Using data from the Framingham Heart Study (FHS) [45] we tested the validity of this model and estimated transmission parameters for various health related behaviors, though the focus here is on obesity as an example. To both demonstrate that obesity can display infectious-disease-like dynamics, and to estimate values for the model parameter 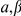, and 
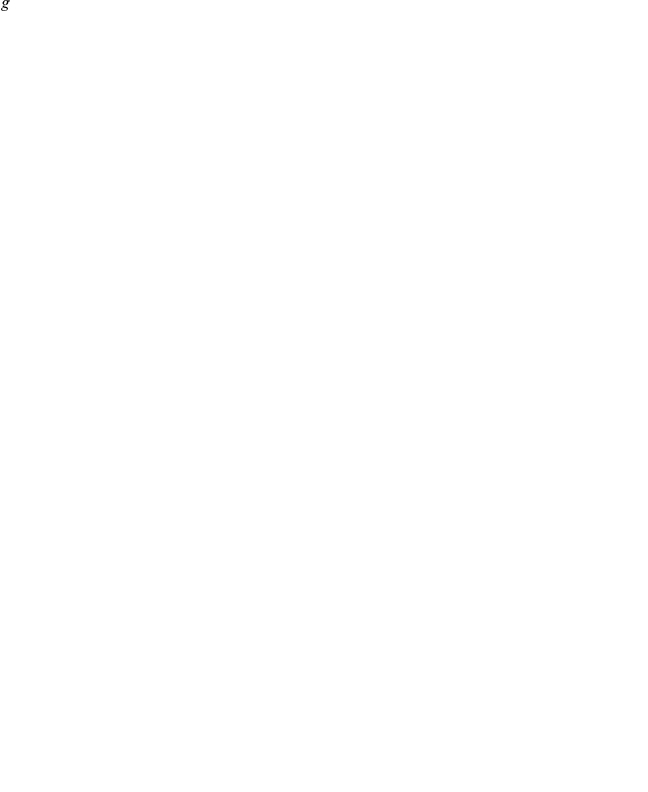
, we use dynamic information about transitions between states based on our multiple time points of data. For data points separated by time intervals (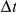) smaller than the average time between transitions, the transition probabilities can be linearized. The probability of a transition from susceptible to infected after a time 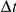 can be given by 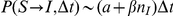, and the probability of transition from infected to susceptible after time 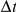, by 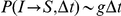. It is necessary for the time between measurements to not be comparable to or greater than the average lifetime of a state to keep the probability of double transitions within a time interval low.

This epidemiological approach to social contagion has important differences from other models which look at correlations in present and past states of connected individuals. Here, similar to others [16], [19], [20], [46] we look at how contacts influence the transitions between states, which better captures the nature of contagion. Since we use pre-existing social ties, we do not see effects from selection bias in choosing friends with similar states. Additionally, time invariant confounding events that lead to concurrent changes in connected individuals will not show up as contagion effects in this model.

The dataset we use is a subset of individuals from the Framingham Heart Study [45]. This study was initiated in 1948 in Framingham, Massachusetts and has continued enrolling subjects through the present. We examined individuals in the Offspring Cohort, enrolled starting in 1971. Subjects come to a central facility at regular intervals (approximately every 4 years) for medical examination and collection of other survey data. Body mass index (BMI) was measured at each exam, and obesity was defined as BMI
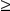
30 [47]. All other, lower, weights, which include underweight, normal range weight and over-weight, were classified as ‘not obese’. In additional to information on mental and physical health, subjects were asked to name at least one close friend at each exam, and were also connected to all first-order relatives, as well as coworkers and residential neighbors. For each subject, the following social connection data is available: (i) each other person to whom they were connected, (ii) the dates of initiation and termination of that relationship, (iii) the type of relationship (neighbour, coworker, first-degree relative, or friend), and (v) the geographic distance between the two subjects. The social network for each exam was constructed by creating a network matrix G, where 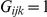 if subject 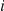 nominated subject 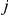 as a connection before or during the time that subject 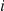 was administered exam 
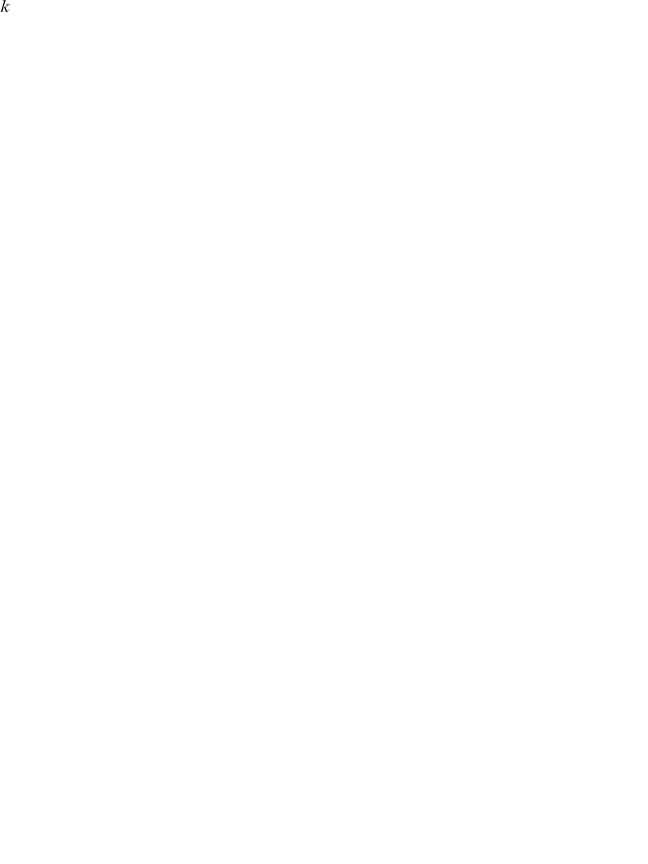
. All relationship types are mutual except for friendships, which are self-nominated, such that 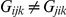 is possible for friendships.

To study the transmission of obesity, we examine changes in BMI between sequential exams. Seven exams were administered to the Offspring Cohort between 1971 to 2001, with network data collected for each. We examine transitions occurring between each exam. The average fraction of the network that was classified as obese increased between these seven exams, suggesting the transmission process is not yet at steady state (Exam 1: 14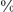 obese; Exam 7: 29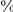 obese). Each set of exams were closely and consistently spaced (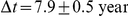 (exam 1), 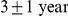 (exam 7)). In general when modeling an infectious process, the rates of infection and recovery are assumed to be constant over time, with the prevalence changing as the infectious process begins and finally reaches equilibrium or is eliminated. When examining the spread of obesity using longitudinal data on transitions between exams, we can actually test this assumption and detect changes in the rates themselves.

A given state 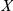 is considered infectious if having more contacts in state 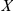 makes you more likely to switch to state 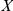. That is, a positive relationship between the number of contacts in state 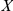 and the probability to transition from state 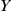 to state 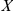 indicates that state 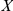 is infectious with respect to state 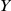. Therefore, to test whether a given state 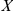 is infectious with respect to another state 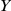, we perform an ordinary least squares (OLS) linear regression as follows. Each subject in state 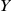 in exam N is coded as either having transitioned to state 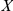 (transition = 1) or not (transition = 0) in exam N+1. We then regress this binary transition variable for each subject against the number of contacts in state 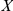 that subject had during exam N. A significant positive correlation indicates that having more friends in state 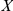 at the earlier exam makes you more likely to switch to state 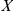 in the later exam. If state 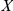 is infectious (a significant positive correlation exists), then the value of 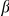 can be calculated from the slope of the regression line, and the value of 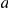 can be calculated from the intercept. If state 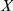 is not infectious (no significant correlation exists), then the value of 
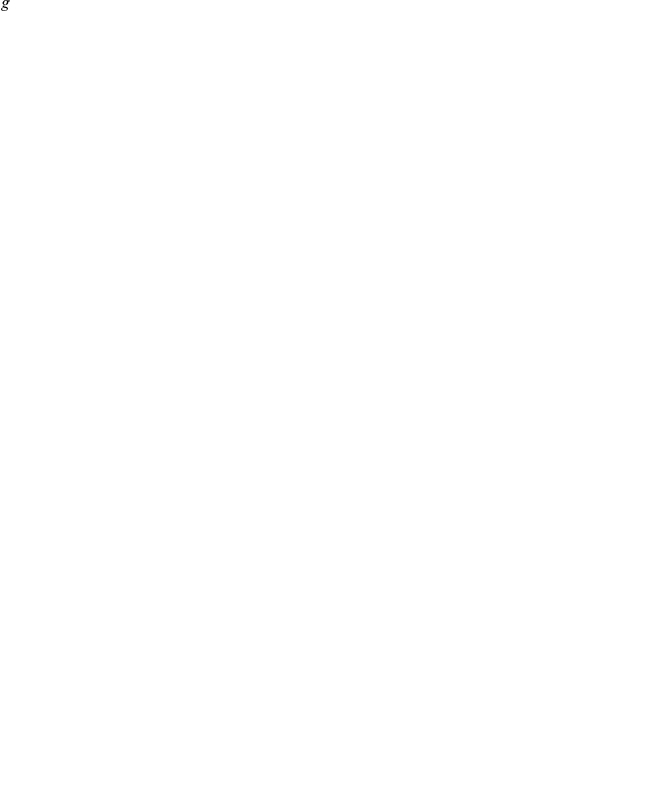
 can be calculated from the intercept. 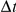 was taken as the average time between examinations, which varied between exams from 3 to 8 years. Using logistic regression as opposed to OLS regression gives very similar results, as the datapoint line is within the linear range of the logistic model.

**Figure 2:**
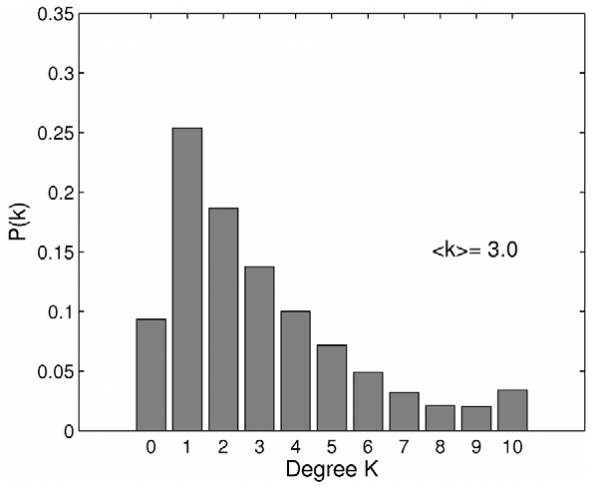
The degree distribution of the Framingham Heart Study Network. The degree distribution of the Framingham Heart Study social network at the most recent exam (7) considered in this study. Connections include friends, family and coworkers. The average degree is around k = 3 and the transitivity is 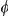 = 0.64 (the ratio of triangles to triples).

The structure of the Framingham Heart Study social network varies over the course of time, ranging from 7500 individuals with an average of 5.3 connections each at the first exam, to 3500 individuals with 2.8 connections on average at the seventh exam. Summary statistics are presented in the supplement (Table S1). These changes in population size and average degree occur because individuals may die or drop out of the study but new individuals are not added. The network is approximately Poisson distributed (see Figure 2), although with some subjects having no connections. The transitivity 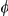 is consistent over time at approximately 0.64. While neighbors were included as contacts in the study, like Fowler and Christakis [20] we find no significant trends when including neighbors, and so did not include these contacts. For friendships, we only consider the contacts of an individual to be those other individuals whom they nominated (other relationships are all mutual), and so the network is directional.

**Figure 3:**
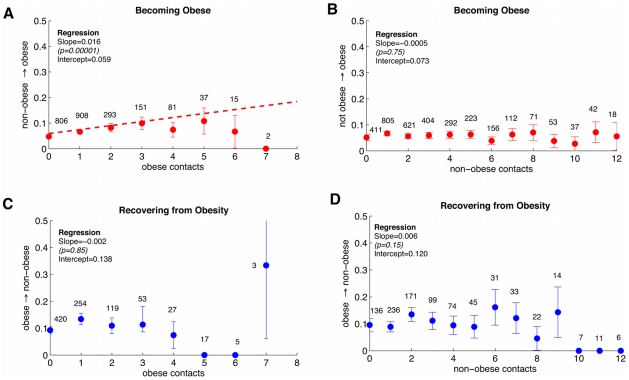
Evidence for disease-like spread of obesity. Obesity behaves like a disease agent, infecting those in a susceptible ‘not obese’ state. The probability of transitioning from ‘not obese’ to ‘obese’ increases in the number of ‘obese’ contacts (A), and doesn't depend on the number of ‘not obese’ contacts (B). Conversely, the probability of recovering to the ‘not obese’ state does not depend on the number of ‘not obese’ contacts (D) or the ‘obese’ contacts (C)). Labels above points on plot are the number of observations averaged into that data point, and error bars are the standard error of the proportion.

**Figure 4:**
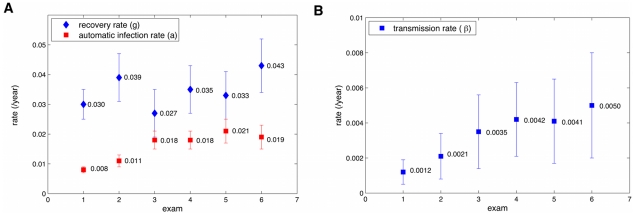
Change in observed parameters over time. Parameter measurements for obesity from each set of consecutive exams. Data point at exam N represents the value for the transition from exam N to N+1. Error bars are 95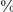 confidence intervals on measurements from regression of transition probability versus number of contacts of a certain type. (A) Contact-independent rates. The rate of recovery (
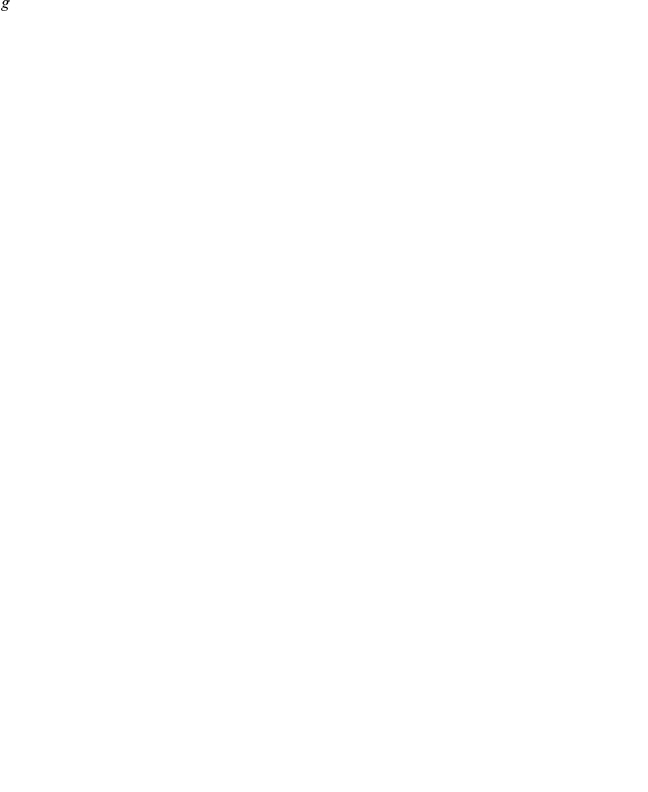
) appears to be constant within the margins of error throughout the study while the rate of automatic infection (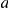) appears to increase between exams 1 and 3, then stay constant. (B) The contact-dependent transmission rate (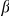) appears to increase over time.

The results of infectiousness analysis for the spread of obesity between exams 4 and 5 are shown in Figure 3 as an example. Consistent with the SISa model formulation, we find a significant positive correlation between the probability of transitioning from ‘not obese’ to ‘obese’ and the number of ‘obese’ contacts (Figure 3A, coeff = 0.016, p = 0.0001), and no significant relationship between the transition from ‘obese’ to ‘not obese’ and the number of ‘not obese’ contacts (Figure 3D, coeff = 0.006, p = 0.15). Additionally we find no significant relationship between the probability of transitioning from ‘not obese’ to obese and the number of ‘not obese’ contacts (Figure 3B, coeff = −0.0005, p = 0.75), or the probability of transitioning from ‘obese’ to ‘not obese’ and the number of obese contacts (Figure 3C, coeff = −0.002, p = 0.85). The same analysis was repeated for each interval between sequential exams and very similar results were found. The full results from the regression analysis are presented in the supplement (Table S2). This suggests that obesity can indeed be modeled as an infectious process in the SISa framework, with ‘not obese’ susceptibles becoming ‘obese’ infecteds, and transmitting obesity to other susceptibles. The parameters for the SISa model can be calculated from the transition probabilities mentioned earlier, by dividing slope and intercept values by 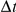, the average time between exams. These values are reported for each exam in Figure 4, and the values at the latest exam interval are summarized in Table 1. For most recent exam, the transmission rate, 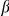, is found to be 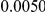/year. The spontaneous transmission parameter 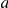 is found to be 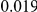/year. The recovery parameter 
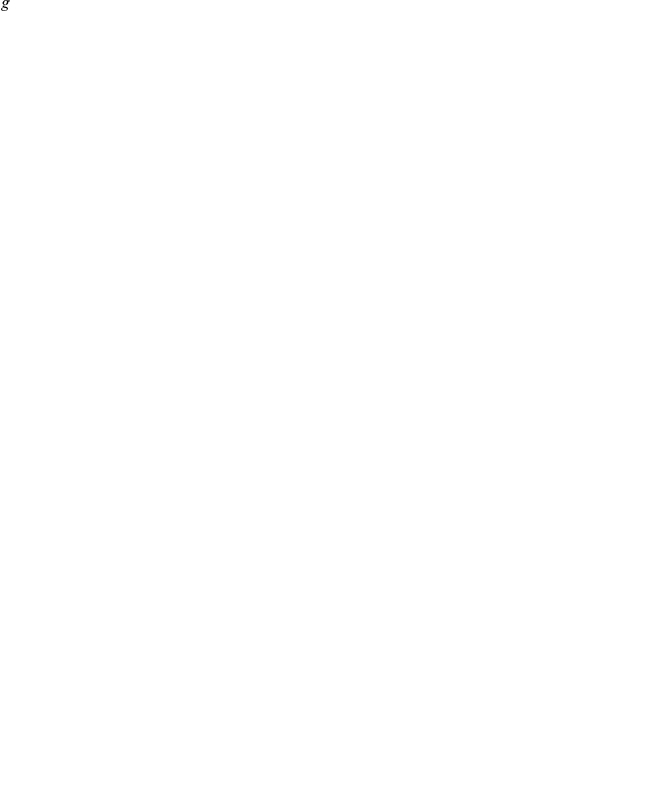
 is found to be 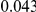/year. From these SISa model parameters, other values of interest can be calculated. The ‘average lifetime’ of a state is the average length of time and individual spends in this state before recovering, which was found to be 24 years for this time period. The ‘influence’ of a state is the cumulative probability that the infection will be passed from an infected to a susceptible connection before the infected individual recovers, and is observed here to be 13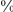. The ‘cycle length’ is the average length of time between spontaneous infections, and is 56 years. The basic reproductive ratio is approximately 0.35, which implies that without spontaneous appearance, the obesity epidemic would not be self-sustaining based on transmission alone. However this calculation is an approximation since uses the formula for a population that is well-mixed but only effectively contacting a fraction of the total population at each time (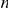 contacts), so does not factor in fixed network structure (there is no analytic formula for this situation). We observed a correlation in the positioning of obese and non-obese individuals of 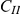 = 1.3 and 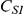 = 0.9.

**Table 1:** Parameter estimates for obesity between exams 6 and 7 (1995–2001) using the SISa model framework.

Parameter	Description	Value
a	rate of spontaneous infection	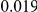 /yr
g	rate of recovery	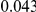 /yr
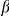	rate of transmission through contact	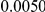 /yr
1/a	cycle	53 years
1/g	lifetime	24 years
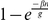	influence	0.13
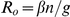	basic reproductive ratio	0.35

The ‘average lifetime’ of a state is the average length of time an individual spends in this state before recovering. The ‘influence’ of a state is the cumulative probability that the infection will be passed from an infected to a susceptible connection before the infected individual recovers. The ‘cycle length’ is the average length of time between spontaneous infections. The basic reproductive ratio is calculated by setting 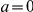. However this calculation is an approximation since it does not factor in fixed network structure. Since 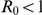, the obesity epidemic would not be self-sustaining based on transmission alone.

Since these rates were measured for 6 different inter-exam transitions over 30 years, we can look at how the value of these rates changes over time. Figure 4 shows the measured automatic infection (a), transmission (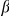), and recovery rates (g) for each exam interval. Error bars are 95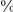 confidence intervals on measurements from analyses like Figure 3. While the rate of recovery (
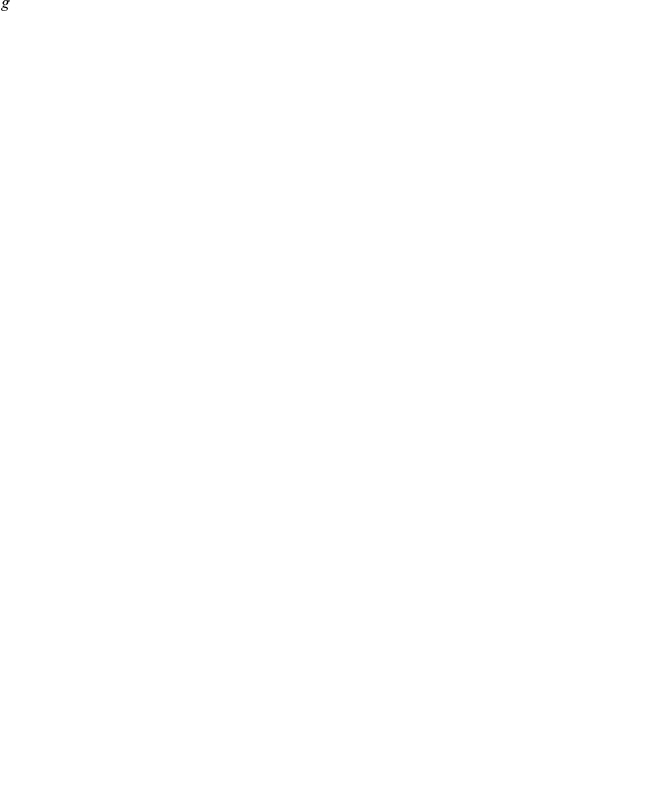
) has remained relatively constant since the 1970s, the rate of spontaneous infection (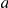) has steadily increased over time. The transmission rate, 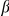, also appears to have increased over time. These trends were tested using weighted regression (to include the different errors for each measurement) and found to be significant for 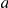 and 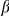 but constant for 
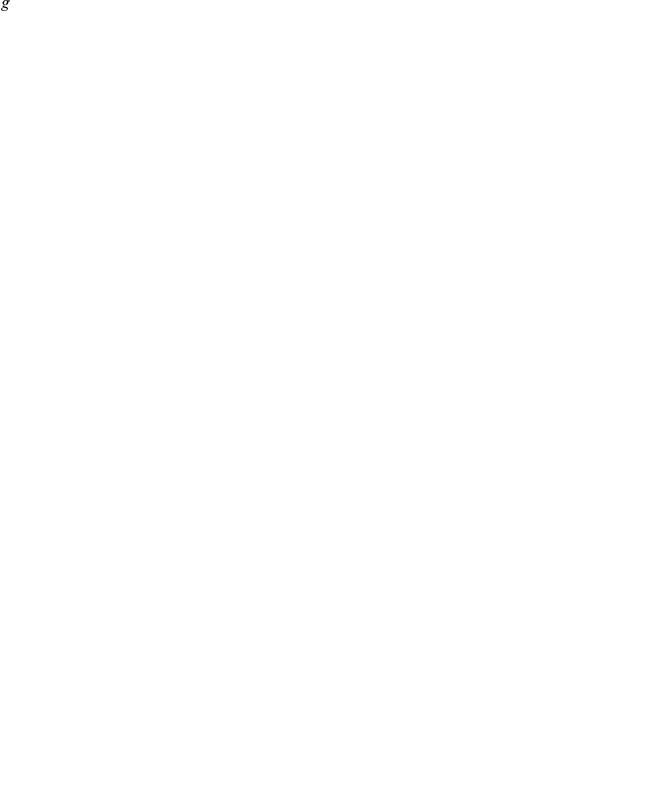
. For the rest of the study we used the time-averaged value of 
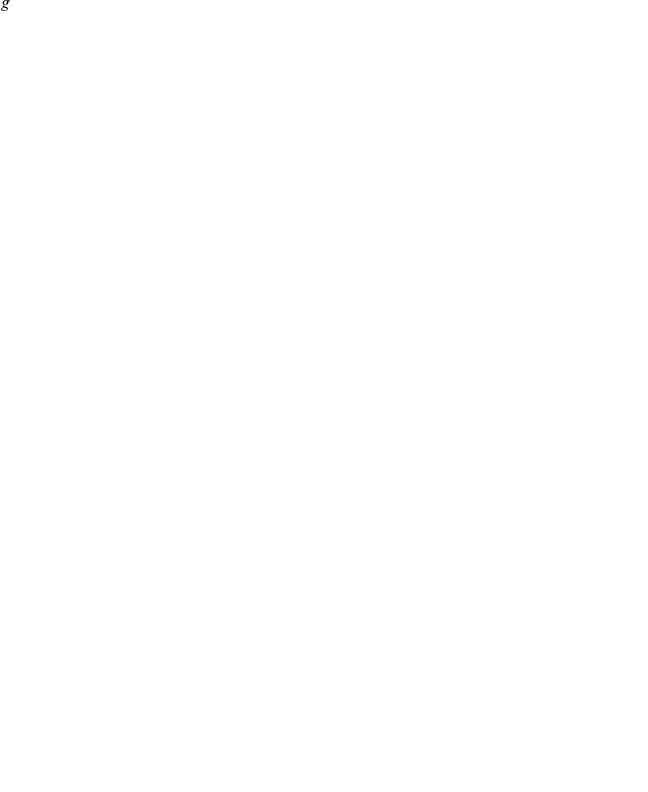
, 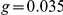. This suggests that the obesity epidemic may be driven by increasing rates of becoming obese, both spontaneously and transmissively, but not by decreasing rates of losing weight.

We also found that both happiness and depression fit the SISa model, both being contagious from a neutral emotional state [18], that smoking cessation, though not smoking itself, also fit, and that both alcohol consumption and abstinence were contagious from the opposite state (data not shown). For all of the above cases, we tested if the transition probability depended instead on the *fraction* of contacts in a state, instead of the *number*, and found no significant dependence. We also tested for dependence on other personal attributes such as age, sex and education, and found no dependence in most cases. For obesity, the transition probability from not obese to obese decreased slightly with age (coeff = −0.0012, p = 0.04). Our results show that many models of social influence make assumptions about interpersonal interactions that are not supported by this longitudinal data. One of these assumptions is the ‘majority rules’ interaction, which assumes that people will be most likely to switch to the state most of their contacts are in [40]. Here, transitions depend on the number of contacts, and only certain states (those we class as ‘infectious’) actually influence transitions (in other words, contagion is only in one direction). This has significant effects on the predictions for epidemic progression. For example, ‘majority rules’ models predict 100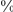 infected at steady state, and that weight loss behavior spreads and so an effective intervention is to ‘pin’ certain individuals at low weights. Also, many models assume that the probability of transitioning to a state is zero if no contacts are in that state, but these results show that there is a constant probability of spontaneously becoming ‘infected’. Finally, using this framework, we can get rates for transitions, and hence have an idea for the time-course of the progression, not just the final outcome.

### Case study: Modeling the obesity epidemic

**Figure 5:**
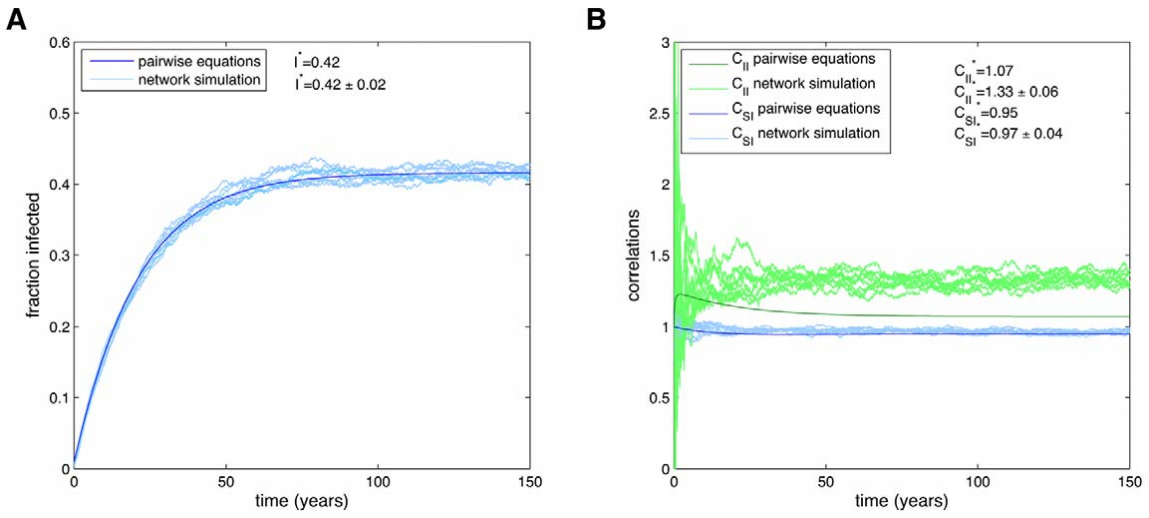
Simulations of obesity epidemic using SISa model. Time series of an epidemic on the Framingham Heart Study network, using full simulations (light blue) or the n-regular pair-wise equations (dark blue). Parameters used are those measured for the obesity epidemic: 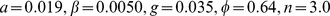. In the SISa model there is a co-existence of susceptible and infected individuals at steady state. For these parameters there is a good agreement with simulations and the pair-wise equations for the fraction infected (A), but the equations predict less correlations (B), due to the neglect of heterogeneities in the number of contacts.

In this section, we will use the SISa model to make predictions and evaluate interventions for the obesity epidemic, using the parameters observed in the FHS data. For simplicity and generality, we will keep the parameters 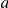 and 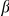 constant at the values observed for the most recent exams, and use the time-averaged value of 
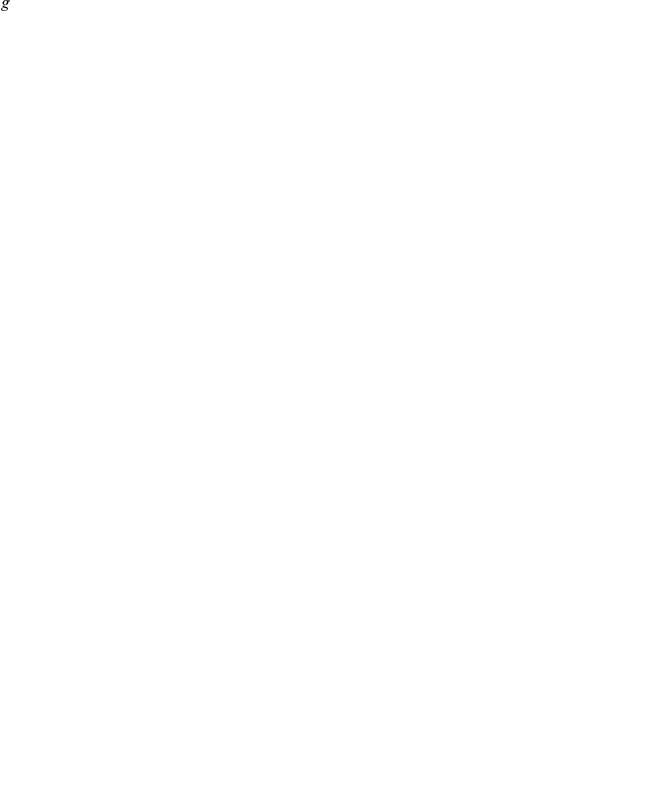
. Since we are mostly interested in predicting future trends, and the parameters seem to have relatively constant values over the final decade, this simplification should not affect these predictions. We also keep the network fixed at the structure observed at Exam 6, except when we compare to historic data. While the simplified pair-wise equations we present are designed for symmetrical networks, they can be approximately adapted to directional networks by letting 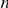 represent the average out-degree (average number of influential contacts) instead of the total number of contacts. In the Framingham data, greater than 90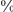 of contacts are symmetrical, and so there is little error in this approximation. For hypothetical networks were the contacts formed by out-degree and in-degree are very different sets of individuals, deviations are expected. Figure 5 shows the results of both the n-regular pair-wise equations and a full simulation on the FHS network for the spread of the obesity epidemic. The parameters used were those measured from FHS as discussed earlier. One of the important properties of the SISa model is that it always leads to a stable coexistence of both infected and susceptible individuals, with infecteds becoming 100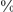 prevalent only in the limit as 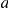 or 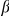 approaches infinity. This is very different from statistical-physics-based interaction models where the population always ‘coarsens’ to everyone in a single state [40]. These results show that for the parameters measured for obesity, the pair-wise equations are not significantly different from the full simulations for predicting prevalence, and hence provide a good substitute. The reason is that the spontaneous rate (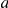) is significantly larger than the transmissive component (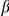). For larger values of 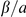, there is a noticeable difference (shown in the next section).

This model predicts that, assuming the rates do not further change over time, the steady state proportion of obese individuals will be 42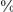. While not great, this is a much more optimistic estimate than 100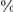 [40]. However, all of the parameters observed in this study have an error associated with them, and so there is some uncertainty in this prediction. Figure 4 shows the ranges of the 95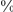 confidence intervals for these values. We can estimate the uncertainty in this prediction by using first the values of these parameters, within the range of one standard deviation, that would give the highest prevalence (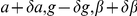) and then those that would give the lowest (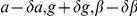). We used 
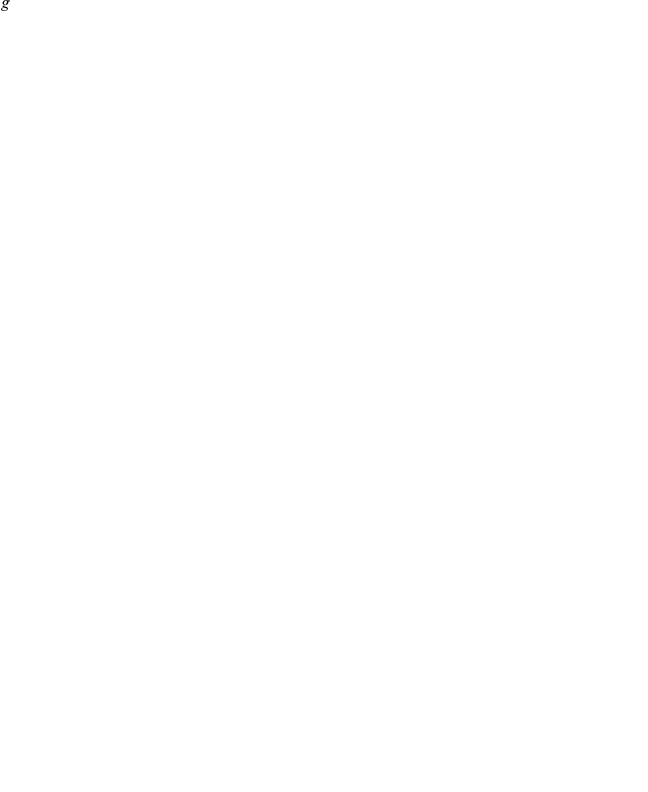
 = 0.05, 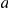 = 0.015 and 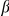 = 0.002 to get the minimum and 
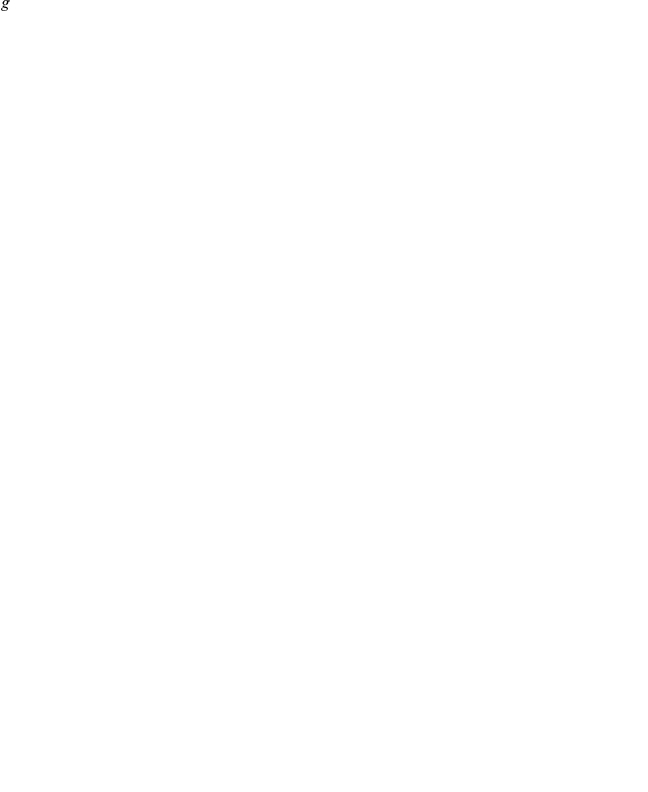
 = 0.03, 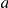 = 0.023 and 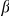 = 0.008 to get the maximum. These simulations suggest the confidence interval for the expected prevalence can be approximated as 25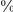 to 54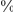. This model also allows us to estimate the time-course of the epidemic, and suggests it would take around 40 more years for the obesity prevalence to be within 1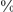 of this maximum value. At the first time point in our data (1970), we measured the rates to be 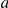 = 0.008, 
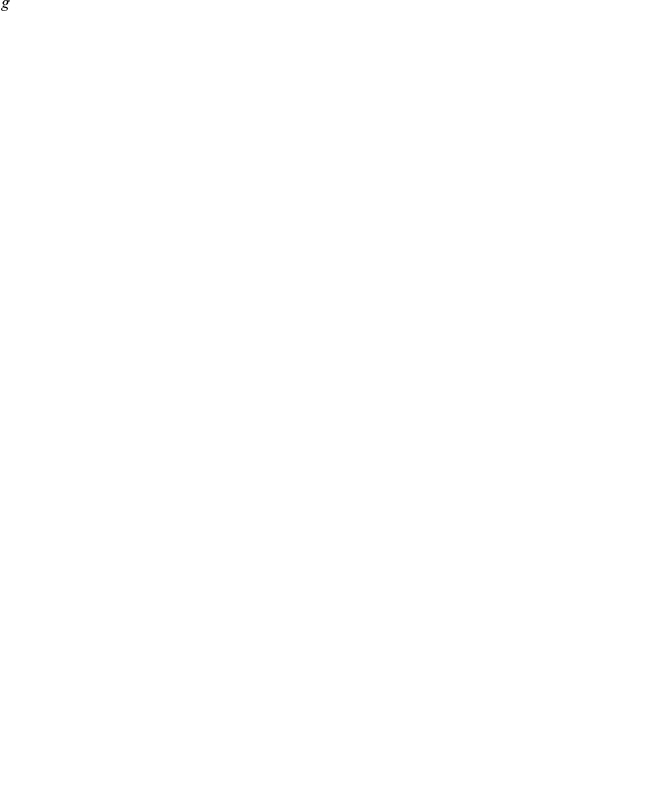
 = 0.03 and 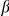 = 0.001, and the prevalence to be 14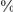. These parameters would have led to a steady state prevalence of 24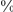, which suggests that the rates of becoming obese must have originally been much lower than this.

**Figure 6:**
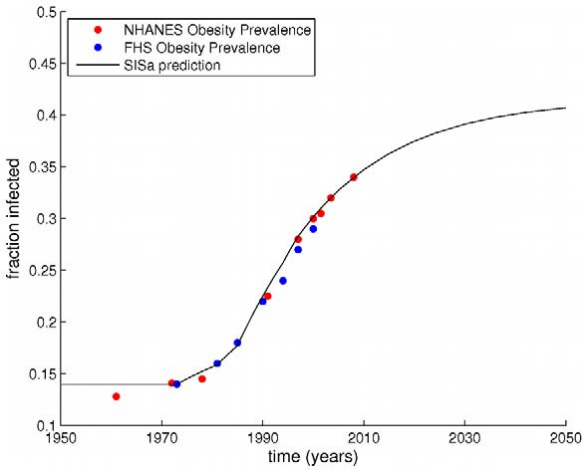
Comparing SISa model timecourse to historical data. A comparison of historical data on the prevalence of obesity in the Framingham Heart Study (blue dots) and the National Health and Nutrition Examination Survey (red dots) with the timeseries predicted from the SISa model with time-varying parameters. For the simulation, we allowed the parameters 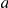 and 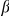 to vary as observed in Figure 4, but kept 
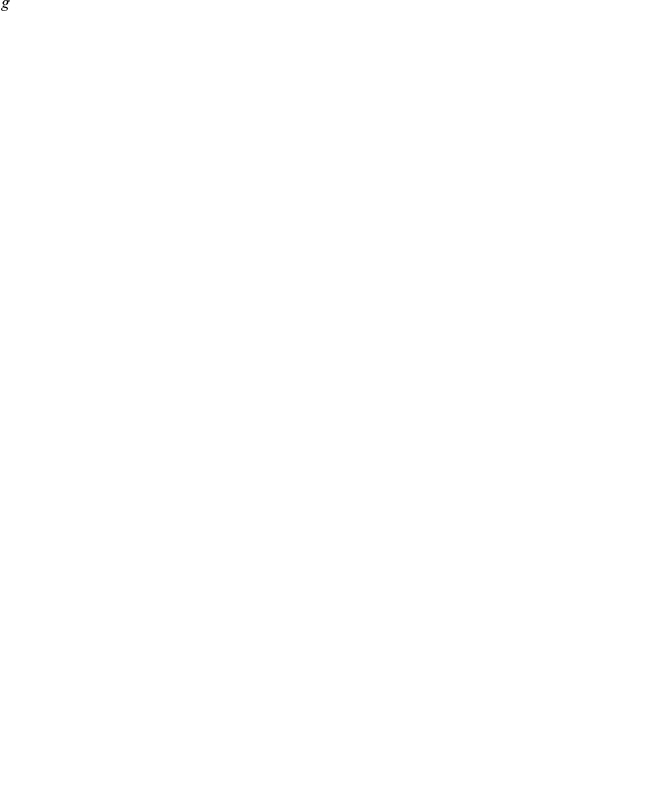
 constant at its average value. Before 1970 (when our measurements started), the prevalence of obesity was assumed to be stable at 14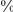. The model and the data both show very similar rates of increase, with a slow post-1970 increase, followed by a rapid increase, and then increasing more slowly. The SISa model predicts the prevalence of obesity will increase slowly to a peak at 42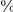.

We can also compare historical data on the obesity prevalence (from both national studies [47] and the FHS data) to the predicted time course shown here. To generate the model prediction, we simulated an epidemic with the pair-wise equations but allowed the rate values and network parameters to change as measured from the data (see Figure 4 and Table S1). We kept 
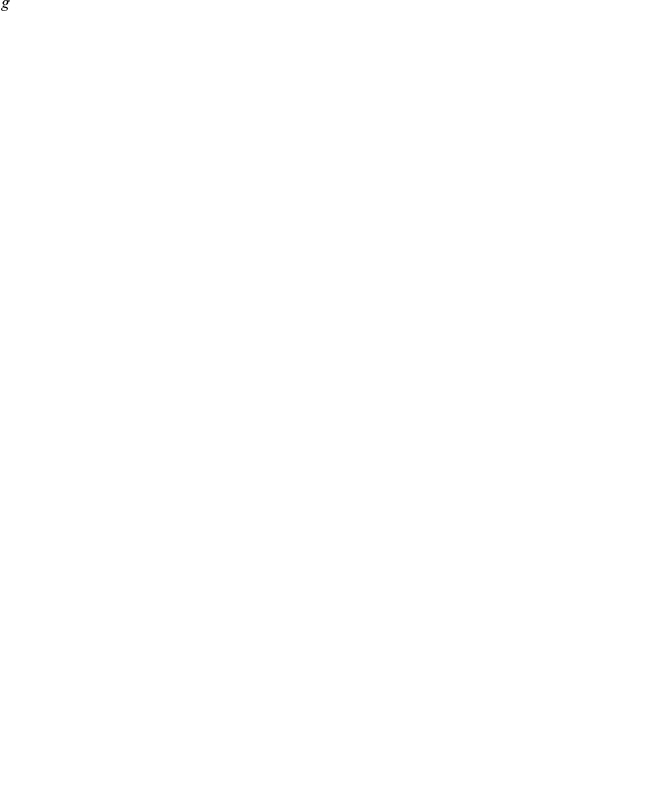
 constant at the average value observed, 0.035, and varied 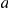 and 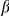 as observed. The value for parameter 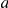 measured for the transition between exam 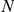 and 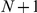 (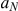) was used in the simulation for times (years) between the average examination dates of exams 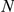 and 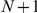, and then increased to 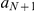 for the next time interval. The same was done for 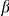. For times before the earliest data points in FHS for which we have measured rate constants(pre 1970), we assumed the epidemic was at a steady state of 14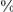. This could be achieved, for example, with 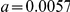 and 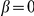. Figure 6 shows that there is a good match in the time course of the model with reality after 1970, with similar rates of increase in the prevalence.

**Figure 7:**
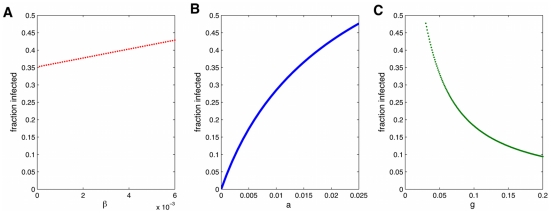
Fraction infected versus SISa model parameters. Dependence of the equilibrium fraction infected on obesity interventions which act to change the rates of infection (transmission (A) and ‘automatic’ infection (B)) or recovery (C). When not varying, parameters are 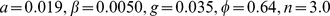.

We can use the pair-wise equations to see how the steady state prevalence depends on various parameters, which is especially useful to see how interventions that aim to change a certain parameter may affect the prevalence. Figure 7 shows these results. For the parameter values for obesity, although 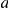 is quite large, 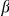 is still important. If 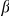 changes from 0 to 0.005, the expected steady state changes from around 0.35 to 0.42. However, much larger changes can be realized by decreasing 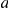 or increasing 
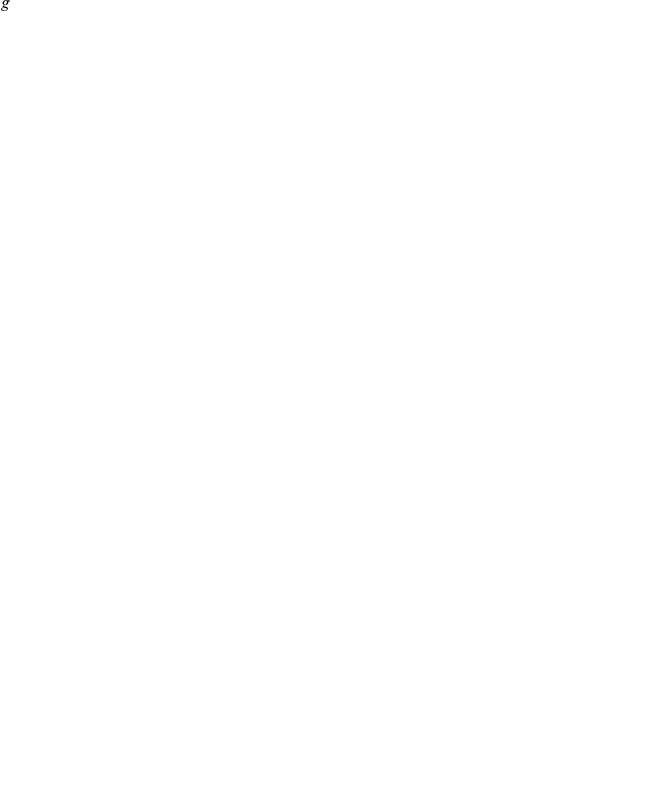
. For the obesity parameters, completely removing the contagious component is only expected to change the steady state prevalence by around 7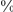. However, changing the spontaneous infection term can have much larger effects. While a 50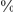 change in 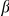 will result in only a 3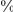 decrease in I, cutting 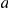 in half will reduce the prevalence by 15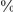. However, a similar absolute decrease of 0.005 would also lead to a 7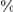 difference. The efficiency of changing one parameter versus the other can be looked directly at 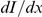 for various parameters, which will be shown in the next section.

### General properties of SISa model

**Figure 8:**
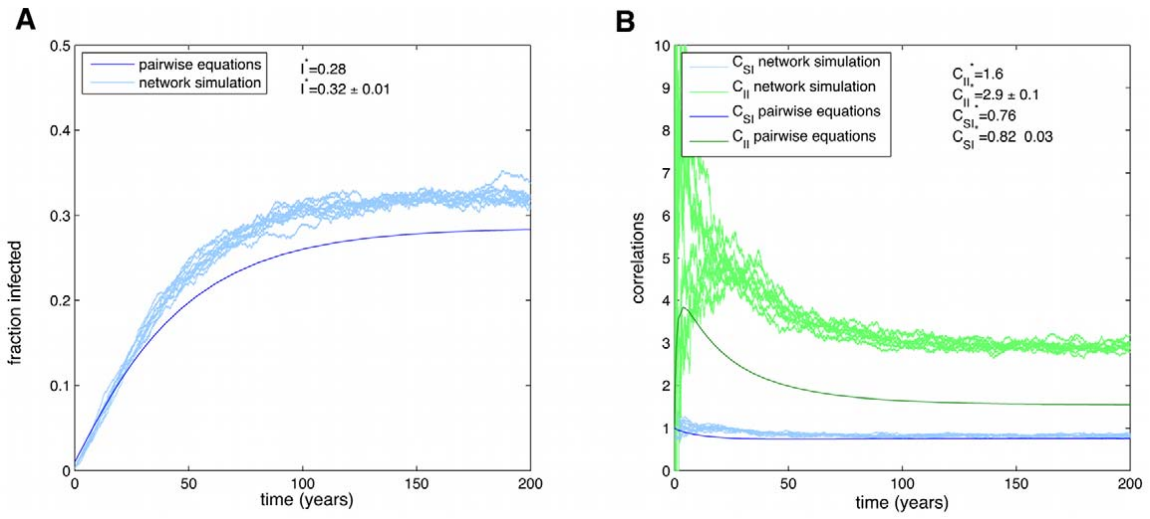
Pairwise equations diverge from simulations when transmission is higher. Time series of an epidemic on the Framingham Heart Study network, using full simulations (light blue) or the n-regular pair-wise equations (dark blue). When the ratio of 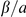 is larger than that observed for the spread of obesity, the pair-wise equations diverge more from the full simulations, both for the fraction infected (A) and the correlations (B). 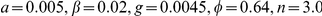.

In this section we will examine the more general properties of ‘infections’ following SISa model dynamics. While Figure 5 showed excellent agreement between the pair-wise equations and full simulations for the time dynamics, this is not true for all parameter regimes. When 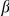 is larger and 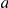 is smaller (as shown in Figure 8), and the network is strongly heterogeneous (as the Framingham network is), the pair-wise model deviates more. The reason is that heterogeneous network effects become more important for larger 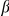, and the pair-wise approximations are best for homogeneous networks. The extension of the pair-wise equations to heterogeneous networks is described in the supplement (Text S1).

**Figure 9:**
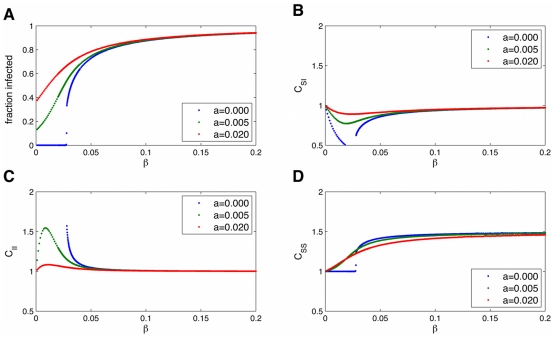
Dependence of the equilibrium fraction infected and correlations on the rate of transmission, 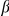. Dependence of the equilibrium fraction infected (A) and correlations (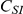:(B), 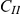:(C), 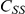:(D)) on the rate of transmission, 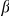. When 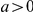, expected in most social infections, there is no longer a threshold (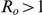) needed for the infection to invade the population. The network causes infected individuals to cluster 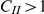 away from susceptible individuals 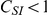, and this is more pronounced for larger 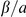 and lower fraction infected. Parameters are 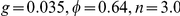.

We can use the pair-wise equations to see how the steady state prevalence depends on various parameters, which is especially useful to see how interventions that aim to change a certain parameter may affect the prevalence. Figure 9 shows how the steady state changes with the rate of transmission, 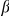. The blue line (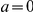) shows what would happen in a classical epidemic, with no spontaneous infection. When 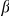 is below a certain value (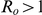), the infection does not spread. The fraction infected increases rapidly with 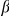 in this regime. As soon as we add 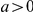, this thresholding behavior disappears. When 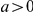 the steady state is less sensitive to 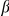. The red line (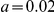) shows the approximate parameter values for obesity. Here although 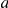 is quite large, 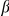 is still important. As with classical infectious disease models [29], disease spread on a network leads to decreased 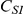, the spatial correlation between infected and susceptible individuals, and increase 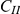 and 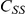, the correlation between pairs of infected individuals and pairs of susceptible individuals, respectively. If we look at 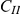, we can see that we expect there to be some correlations of infected people at some 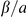 values, but not all. So while seeing spatial correlation may hint there is a inductive process, it is definitely not necessary. You can have an infectious process without seeing correlations, just like you can see correlations without it being caused by the dynamics of influence. Spatial correlation is much higher when 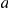 is small.

**Figure 10:**
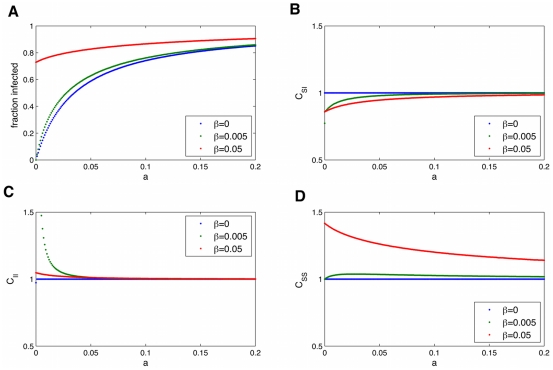
Dependence of the equilibrium fraction infected and correlations on the rate of automatic infection, 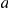. Dependence of the equilibrium fraction infected (A) and correlations (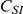:(B), 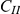:(C), 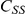:(D)) on the rate of automatic infection, 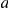. Parameters are 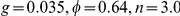.

**Figure 11:**
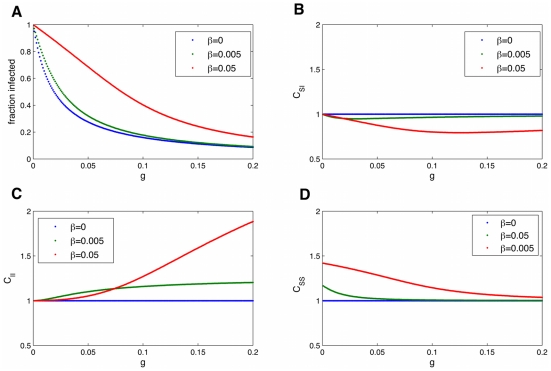
Dependence of the equilibrium fraction infected and correlations on the rate of recovery from infection, 
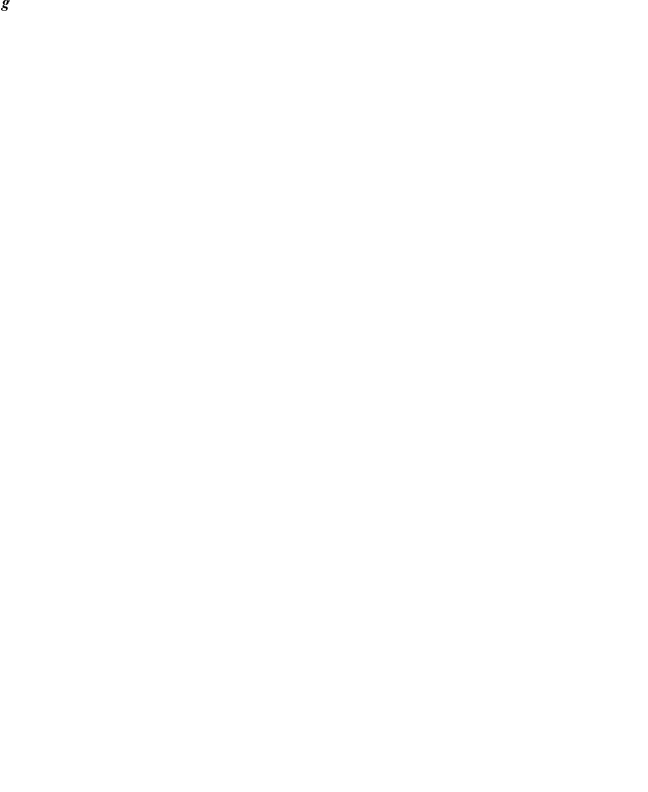
. Dependence of the equilibrium fraction infected(A) and correlations (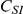:(B), 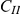:(C), 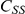:(D)) on the rate of recovery from infection, 
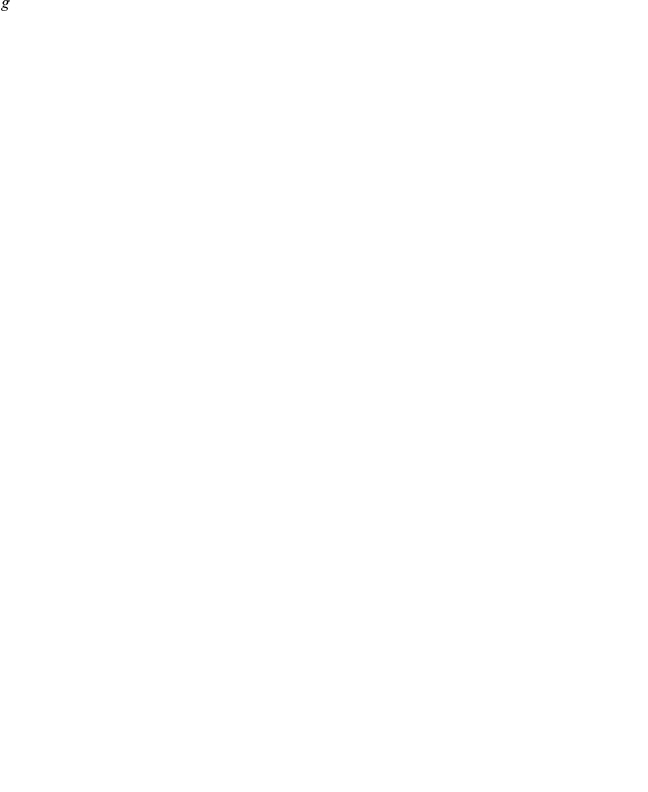
. Parameters are 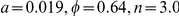.

Figure 10 shows the dependence on the rate of spontaneous infection, 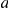. The more spontaneous infection, the more infected. When 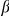 is larger (red line), increasing 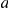 has less effect. The green line is for the parameters measured for obesity. We can use these graphs to compare the effects of various interventions which may reduce the rate of infection. In Figure 9 (vs 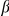), we can see the expected decrease in the prevalence of the infection for a given decrease in 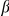. Changing 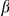 has more effect when 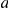 is small. The rate of recovery from an infection is 
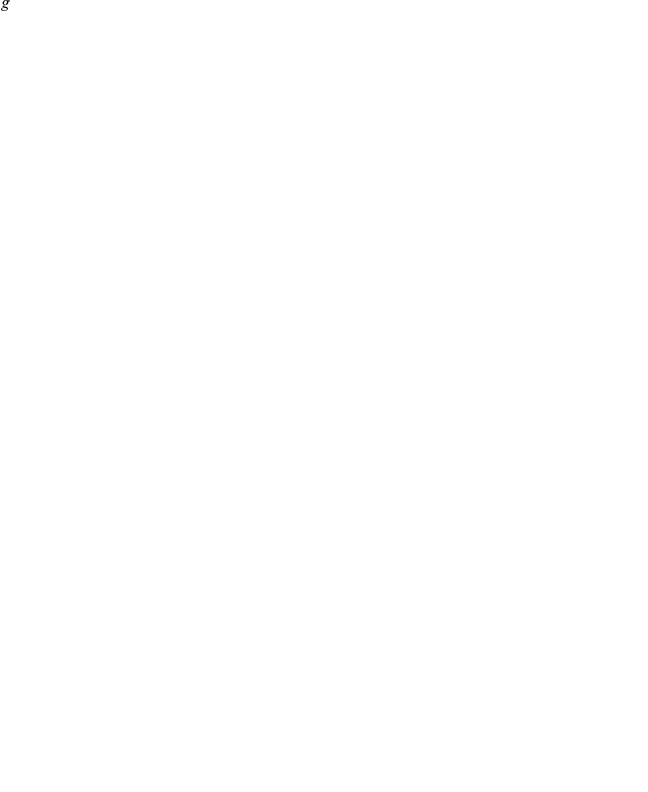
, and in the obesity case, represents the rate at which obese people lose weight and transition to normal BMI values, in probability per year. Higher rates of recovery lead to lower fraction infected (Figure 11). One possible intervention is to increase the rate of recovery. For low recovery values, this has a large effect on 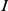, but for 
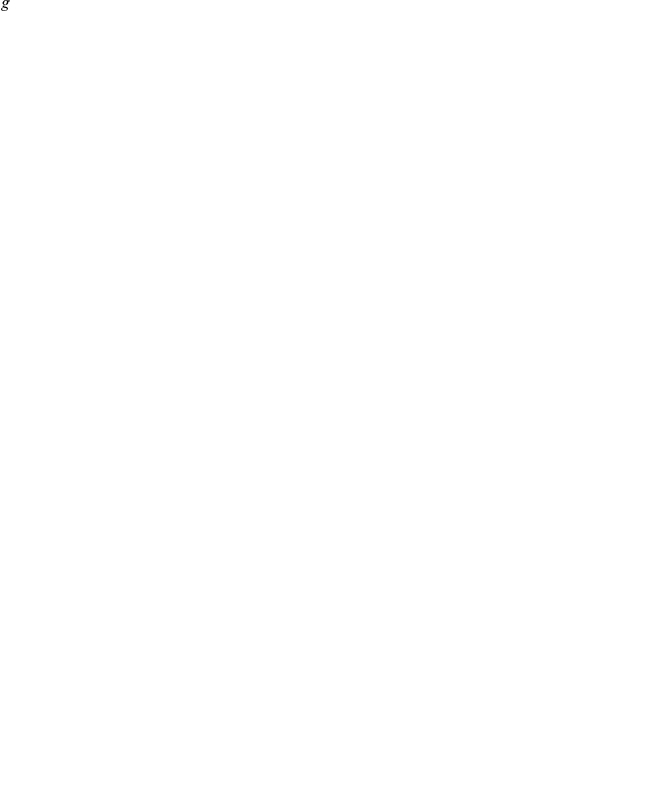
 around 0.04 (the value for obesity), only small changes in 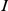 result from changing 
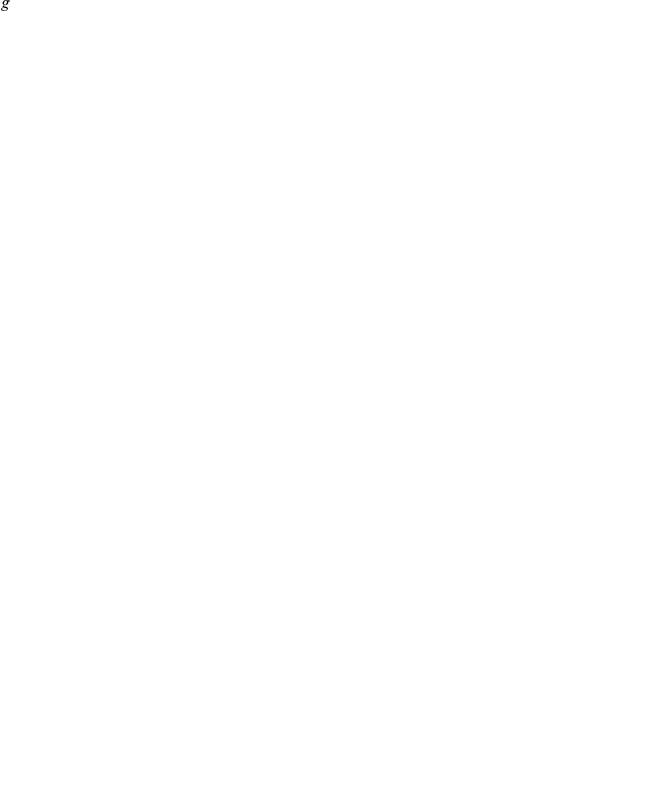
.

In general, the spatial correlations (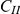) are negatively correlated with the fraction infected (I); more correlations are observed when a disease is not too infectious. If the spatial correlations were fixed to be a certain value (for example obese people cluster together due to selection bias in friendships or confounding factors), then this would actually serve to slow infection. Since we do not observe contagion of losing weight, it does not seem like it would be beneficial to have an intervention which broke up obese clusters.

**Figure 12:**
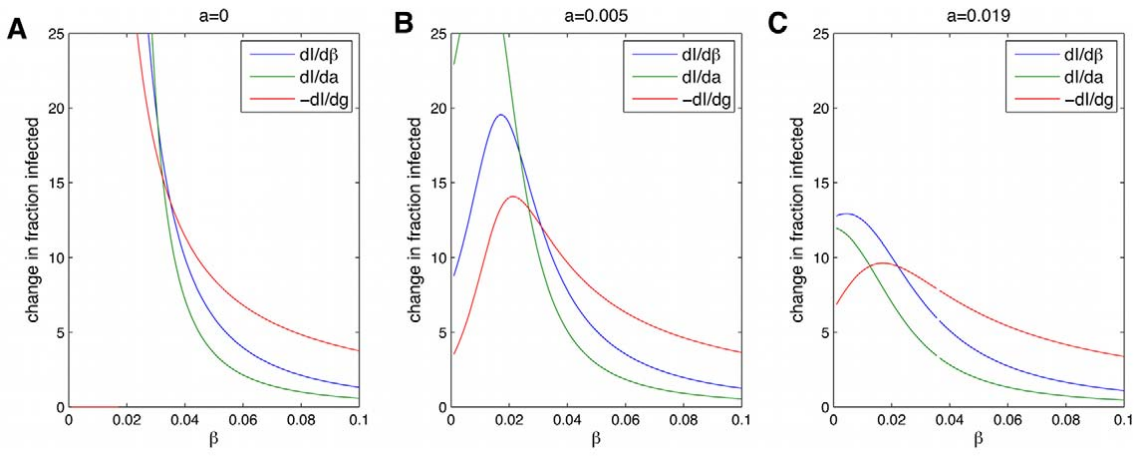
Determining the best parameter to target in an intervention. This graph compares interventions which act to change different parameters of infection (transmission (A), ‘automatic’ infection (B), recovery (C)). Shown is the rate of change of the fraction infected at equilibrium with respect to a change in various parameters of infection. The y axis labels represent the absolute change in the percent infected for a change of 0.01 in one of the parameters. Changing 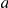 is better for small 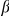 and changing 
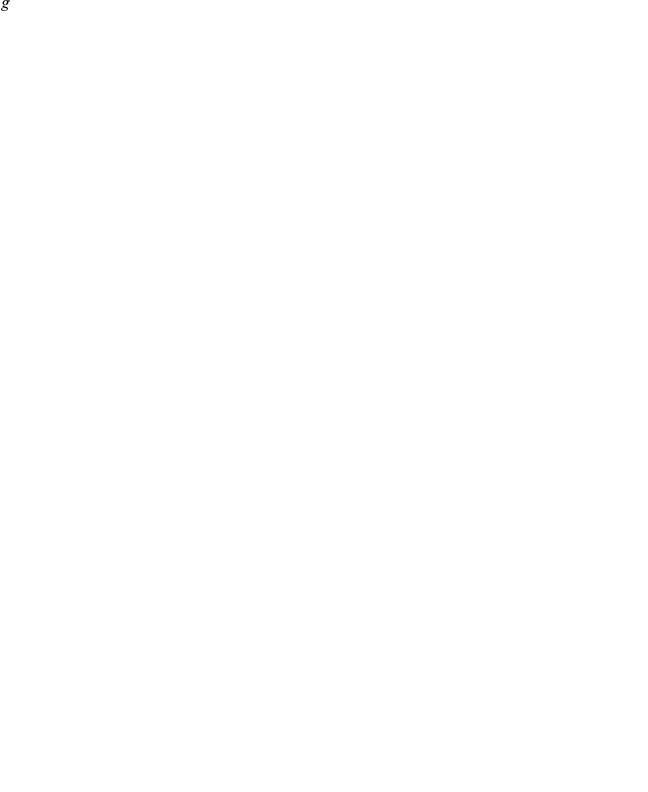
 is best for larger 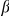. For intermediate 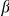, changing 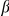 is best. Parameters are 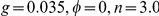.

The most direct way to compare various parameters for spread, and therefore interventions that reduce one of the parameters, is to look directly at 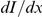 for various parameters (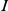 is the steady state fraction infected, 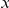 is the parameter of interest. Figure 12 shows that for most parameter regimes, it is always best to increase the recovery rate, 
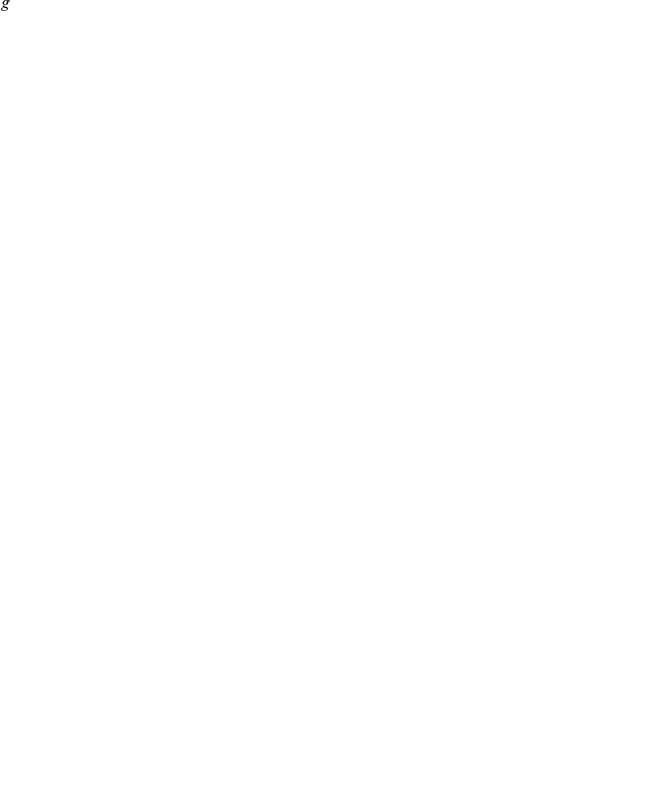
, as a method to reduce the fraction infected, 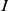. However, for low 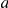 and low 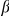, it is best to decrease the spontaneous infection term 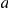, and for a window of intermediate 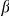, it is best to decrease the transmissive component 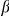. The third plot shows the results for the 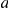 value measured for obesity, and because 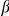 is low here we are in a regime where it decreasing 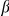 has the most effect, so this is the best intervention.

**Figure 13:**
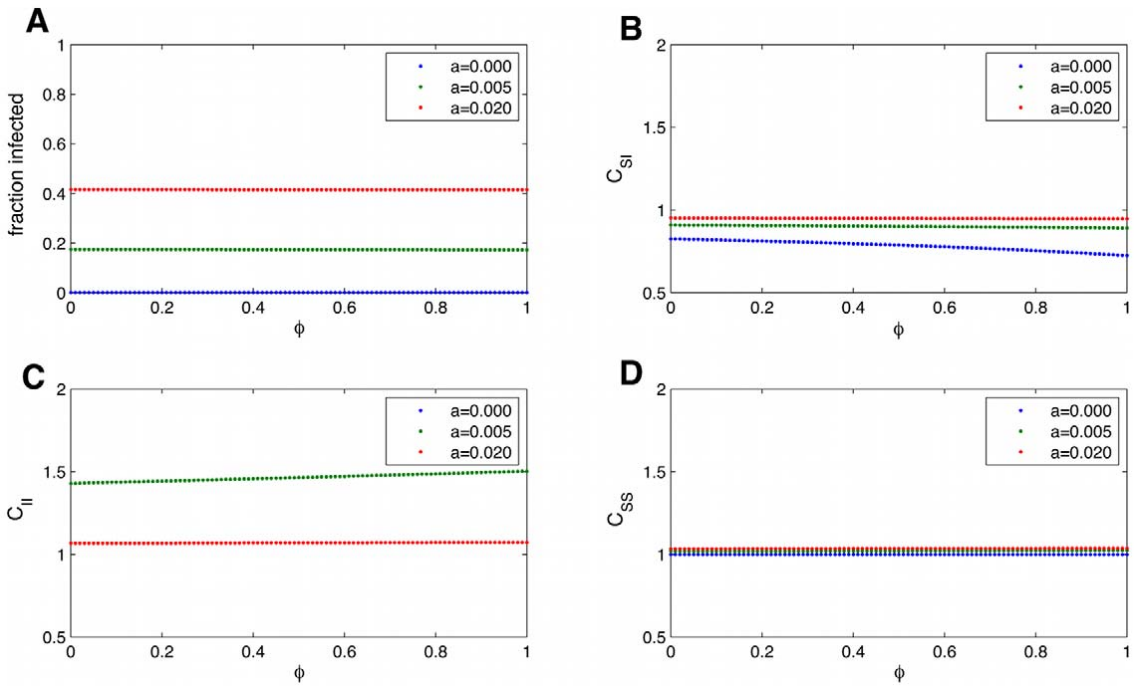
Dependence of the equilibrium fraction infected and correlations on the network transitivity, 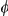. The dependence of the equilibrium fraction infected(A) and correlations (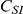:(B), 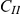:(C), 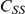:(D)) measured from the pair-wise equations on the network transitivity, 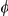. For the parameters measured for the transmission of obesity, shown here, there is no strong dependence on 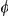. Hence for studying the obesity epidemic it is justified to ignore 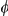 to simplify calculations. Parameters are 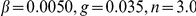.

**Figure 14:**
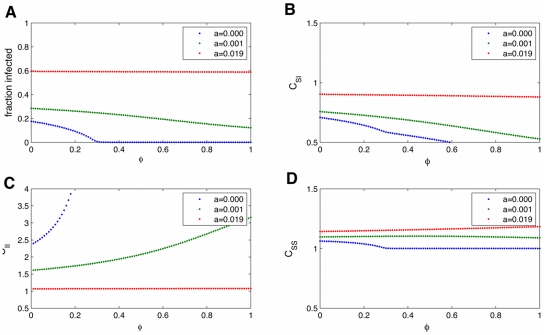
Dependence on network transitivity, 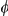, for larger transmission rates. The dependence of the equilibrium fraction infected (A) and correlations (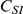:(B), 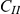:(C), 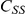:(D)) measured from the pair-wise equations on the network transitivity, 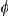. For larger 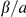, 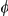 slightly decreases the fraction infected by leading to more spatial correlation of infected individuals. Parameters are 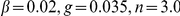.

Many analytic models of network phenomenon assume the transitivity, 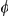, is zero, meaning there are no triangles in the network. This is done to get the analytic expression presented here (Eq. 2), which is not necessary to numerically integrate the pair-wise equations, as presented in the results above. In the FHS network, we observed that 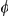 is 0.64, suggesting human social networks are quite transitive. We want to examine the importance of 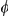 in predicting the fraction infected. For the observed 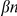 value, the effect of 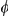 is negligible, as shown in Figure 13. The reason is that the dominant effect here is the spontaneous infection, which does not depend on the network structure. This justifies ignoring 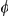 for infections that have only low infectivity terms. However, for large 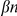 values (the equivalent of 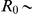
2 is shown in Figure 14) 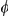 has a more pronounced effect. While for a purely infectious process (blue line), at high 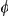, a disease can die out, even for 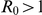, when 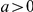, this doesn't occur, but 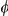 still slightly reduces the spread. It also results in more observed spatial correlation of infected individuals. Overall, there is very little effect of 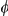 in the SISa model.

We've already discussed how changes in parameters of infection affect the steady state prevalence, and we can consider this an analysis of different types of public health interventions that change rates of recovery, infection or network structure. In previous analysis of the obesity epidemic done by Bahr et al [40] they suggest a strategy of ‘pinning’ groups of people to stay in a non-obese state, similar to vaccinating against an microbial disease, as a method to remove the ‘infection’ from the population. However, in the Bahr model this intervention works (if enough people are ‘pinned’) because becoming non-obese is also contagious, which we don't find in this analysis. In the classical infectious disease setting, vaccinating can lower 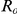 below the threshold for disease invasion, but in the SISa model there is no threshold, and so neither mechanism makes this an effective strategy in the SISa model. Two other possible intervention strategies come out of this model. Firstly, from Eq. 7 we can see that the fraction infected decreases with 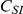, the correlation of susceptible and infected people. If an intervention actively reduced this number, by isolating or clustering infected people, this could reduce the prevalence. Secondly, the fraction infected could be reduced if it were possible to make the ‘susceptible’ state also contagious through contacts.

## Discussion

The SISa model offers a framework for quantitatively analyzing and predicting the public health affects of socially contagious phenomenon. Using a longitudinally measured health outcome and social network data, the SISa model can be used to determine the dynamics of a health trend in terms of rates of acquisition, recovery and inter-personal transmission. From these rates, the relative importance of social contagion can be determined, and changes in prevalence over time can be predicted. The framework can also be used to examine how these rates themselves change over time, helping to understand the mechanisms behind drastic changes in disease prevalence, such as in the obesity epidemic current effecting the United States. Finally, understanding the dynamics of a health behavior using the SISa model allows us to evaluate the benefits of various interventions, especially those that may work within social networks.

The prevalence of obesity in the Framingham Heart Study cohort has increased from 14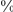 in the 1970s to 30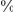 in 2000, and continues to increase. We find that the most recent rate of becoming obese is 2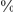 per year and increases by 0.5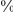 for each obese social contact. The rate of recovering from obesity is 4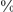 per year, and does not depend on the number of non-obese contacts. These results show that obesity has an infectious character: obesity can be acquired through social contagion as well as through non-social factors. Examining over 30 years of data, we find that these rates have changed throughout the course of the study, with the rate of becoming obese through mechanisms other than social contagion increasing approximately twofold since 1970, and the rate of transmission increasing approximately fourfold. The rate of recovery, however, has changed little. These results suggest that social norms are changing the propensity for becoming obese by non-social mechanisms, and also magnifying the affect that obese individuals have on their non-obese contacts. It is possible that while causing changes in prevalence, these rates may also be responding to changing prevalences (i.e. more obese people leads to increased social acceptability of obesity, which leads to higher rate of becoming obese), creating a positive feedback mechanism and a continuously increasing obese fraction of the population. It has been suggested that changing social norms that stigmatized smoking may have lead to its decline [48], and just the opposite may be true for obesity [49].

Using the SISa model with these parameter values estimated for obesity, we can make predictions about the future of the obesity epidemic and the important factors controlling it. Our models suggest that if the most recent rates stay constant, the population will stabilize at 42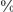 obese. However, it is very likely that the rates of obesity infection may continue to increase if successful interventions are not conducted. Our results show that while the rate of automatic development of obesity appears to have leveled off in the past decade, the rate of transmission has been steadily increasing.

This model allows us to can predict how much spatial correlation is expected from a purely infectious process, and compare this to what is observed in the data, which could be influenced by confounding factors and selection bias in choosing friends. A coefficient of 1 indicates that arrangement of infected nodes is random, while higher values are indicative of spatial correlations. We observed a correlation coefficient for obese individuals of 1.30, which was quite close to what was predicted from epidemic simulations (1.33). This suggests that infection alone is sufficient for explaining the observed correlations, and there may not be much selection bias or confounding factors in effect. We also show that network transitivity is not predicted to have a strong affect on prevalences when there is an automatic component to infection. However, our model also shows that contrary to popular belief, a contagious process on a network does not always result in clustering of infected individuals. This is especially true if there is a large automatic infection term, which is likely with many trends and behaviors.

The SISa approach allows us to compare the effectiveness of different classes of intervention. For the parameter range observed, we find that decreasing the rate of transmission 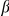 is the most effective intervention (largest decrease in prevalence per unit decrease in rate), although decreasing the automatic infection 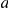 is almost as effective. More generally, while we find that gaining weight is contagious, we do not find that losing weight is contagious. Thus it does not seem to be beneficial to ‘break-up’ clusters of obese individuals or ‘pin’ the weight of certain people in these clusters. Our results actually suggest that clusters of obese people serve to slow the spread of obesity by reducing social contagion to non-obese others outside of the clusters. Another possible intervention would involve somehow facilitating the social spread of becoming non-obese (losing weight), creating a bi-directional transmissive process.

One possible limitation of this study is the incompleteness of the social network dataset used. Because the Framingham Heart Study was not designed as a study of social networks, no attempt was made to capture all of a person's important social contacts. Many close friends of a person could be missing (usually only one friend per person was recorded) and family and coworkers who play only a small part in ones actual social network may have been counted. However, even if under-sampling of real-world contacts did occur in the FHS Network, it does not change our results qualitatively: our data clearly show that rates of becoming obese increase with the number of ‘infected’ contacts (i.e. is contagious) while the rate of ‘recovery’ to a non-obese state does not depend on contacts. However, under-sampling could quantitatively effect our measurement of the rate constants. If a constant number of contacts for each person were missed, our estimate of the y intercept of the transition graphs would be shifted up from its true value, and the actual 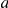 would be smaller than the 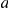 we measured. If a constant fraction of contacts for each person were missed, then our estimate of the x axis would be compressed from its true value and the slope would be increased, so then the actual value of 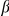 would be smaller than the 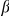 we measured. While it is likely that the FHS network underestimates the total number of contacts, the relationship to the number of ‘influential’ contacts is unclear. In this sense, the observed value of the transmission rates, 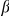, are network dependent. Additionally, network connections may be weighted differently according to their ability to transmit behaviors. Longitudinal studies designed specifically with the intent of measuring social networks and health, which carefully define contacts, such as by amount of time spent together per day, influence, etc, are an important area for future research.

It has recently been suggested that certain, particular types of latent homophily, in which an unobservable trait influences both which friends one chooses and current and future behavior, may be impossible to distinguish from contagion in observational studies and hence may bias estimates of contagion and homophily [50]. The circumstances under which this is likely to be a serious source of bias (e.g., whether people, empirically, behave in these sorts of ways), and what (if anything) might be done about it (absent experimental data of the kind that some new networks studies are providing [22]) merits further study. Observational data invariably pose problems for causal inference, and require one set of assumptions or another to analyze; the plausibility of these assumptions (even of standard ones that are widely used) warrants constant review.

The SISa model as presented here assumes that all individuals have the same probability of changing state (though not everyone will actually change state within their lifetime). It is clearly possible, however, that there is heterogeneity between individuals in these rates. We do not have sufficient data on obesity in the Framingham dataset to explore this issue, which would require observing numerous transitions between states for each individual. Exploring individual differences in acquisition rate empirically is a very interesting topic for future research, as is extending the theoretical framework we introduce to take into account individual differences.

The results we have presented here reiterate an important general principle of network processes: networks tend to magnify whatever they are seeded with, but they must be seeded with something. The increase in obesity is not purely a network-diffusion phenomenon. Automatic infection serves to start and continuously seed the epidemic. Here we show that the dominant process in the increasing prevalence of obesity is contact-independent weight gain; however, the rate of interpersonal transmission contribute significantly to the overall prevalence and appears to be increasing steadily over time. Thus consideration of social transmission and network effects is an important issue for health and policy professionals.

## Supporting Information

Table S1 Summary statistics for the Framingham Heart Study network at each exam. Out-degree is the number of contacts named by an individual. Total degree includes both those who named an individual and those who were were named by an individual. Only friendships are directional, other contacts are symmetrical. Phi (ϕ) is the transitivity of the network. C_SI_ and C_II_ are the spatial correlations between susceptible and infected, and infected, individuals, respectively. N is the number of people for whom both social network and obesity data was available for at a given exam.
(0.01 MB PDF)

Table S2 Summary of results from regression of probability of transitioning between states and the number of contacts in a given state, similar to those shown in Figure 3. n = non-obese, o = obese. The probability of transitioning from ‘not obese’ to ‘obese’ increases in the number of ‘obese’ contacts (A), and doesn't depend on the number of ‘not obese’ contacts (B). Conversely, the probability of recovering to the ‘not obese’ state does not depend on the number of ‘not obese’ contacts (D) or the ‘obese’ contacts (C)). After dividing by the time between exams, the slope of (A) gives β, the intercepts of (A) and (B) give a, and the intercepts of (C) and (D) give g.
(0.01 MB PDF)

Text S1 Deriving pairwise network equations for heterogeneous networks.
(0.15 MB PDF)
