# Supplementary material for: Infectious Disease Modeling of Social Contagion in Networks
Source: PLoS Comput Biol. 2010 Nov 4;6(11):e1000968. doi: 10.1371/journal.pcbi.1000968 (PMC2973808; doi:10.1371/journal.pcbi.1000968)
Supplement: Table S1 — Summary statistics for the Framingham Heart Study network at each exam. Out-degree is the number of contacts named by an individual. Total degree includes both those who named an individual and those who were were named by an individual. Only friendships are directional, other contacts are symmetrical. Phi (ϕ) is the transitivity of the network. CSI and CII are the spatial correlations between susceptible and infected, and infected, individuals, respectively. N is the number of people for whom both social network and obesity data was available for at a given exam. (0.01 MB PDF) [file pcbi.1000968.s001.pdf]

EXAM	out-degree	total degree	phi	C_{SI}	C_{II}	N
1	5.29	5.29	0.68	0.93	1.28	7518
2	4.63	4.69	0.64	0.92	1.18	5608
3	4.07	4.14	0.62	0.92	1.25	4960
4	3.77	3.84	0.61	0.94	1.20	4861
5	3.37	3.44	0.62	0.92	1.28	4415
6	3.00	3.07	0.64	0.92	1.31	3969
7	2.77	2.84	0.65	0.90	1.30	3591
