# Supplementary material for: Infectious Disease Modeling of Social Contagion in Networks
Source: PLoS Comput Biol. 2010 Nov 4;6(11):e1000968. doi: 10.1371/journal.pcbi.1000968 (PMC2973808; doi:10.1371/journal.pcbi.1000968)
Supplement: Table S2 — Summary of results from regression of probability of transitioning between states and the number of contacts in a given state, similar to those shown in Figure 3. n = non-obese, o = obese. The probability of transitioning from ‘not obese’ to ‘obese’ increases in the number of ‘obese’ contacts (A), and doesn't depend on the number of ‘not obese’ contacts (B). Conversely, the probability of recovering to the ‘not obese’ state does not depend on the number of ‘not obese’ contacts (D) or the ‘obese’ contacts (C)). After dividing by the time between exams, the slope of (A) gives β, the intercepts of (A) and (B) give a, and the intercepts of (C) and (D) give g. (0.01 MB PDF) [file pcbi.1000968.s002.pdf]

	EXAM:	1->2	2->3	3->4	4->5	5->6	6->7
A: n->o vs o	slope	0.009	0.01	0.013	0.016	0.015	0.015
	p	0.001	0.003	0.001	0.000067	0.001	0.001
	intercept	0.56	0.053	0.059	0.059	0.071	0.049
B: n->o vs n	slope	-0.0004	0.0004	-0.002	-0.0005	-0.001	-0.002
	p	0.55	0.64	0.17	0.75	0.58	0.49
	intercept	0.064	0.059	0.074	0.073	0.085	0.063
C: o->n vs o	slope	-0.012	-0.009	0.018	-0.002	-0.006	-0.017
	p	0.28	0.489	0.077	0.845	0.446	0.04
	intercept	0.25	0.197	0.102	0.138	0.131	0.132
D: o->n vs n	slope	-0.0001	0.0003	0.007	-0.006	0.001	-0.007
	p	0.97	0.937	0.04	0.146	0.844	0.183
	intercept	0.233	0.188	0.096	0.12	0.123	0.127
