# Supplementary material for: Infectious Disease Modeling of Social Contagion in Networks
Source: PLoS Comput Biol. 2010 Nov 4;6(11):e1000968. doi: 10.1371/journal.pcbi.1000968 (PMC2973808; doi:10.1371/journal.pcbi.1000968)
Supplement: Text S1 — Deriving pairwise network equations for heterogeneous networks. (0.15 MB PDF) [file pcbi.1000968.s003.pdf]

Infectious disease modeling of social contagion in networks

Alison L. Hill, David G. Rand, Martin A. Nowak , Nicholas A. Christakis

Supplementary Info 3

Deriving pairwise network equations for heterogeneous networks

In the main text, the pairwise equations were derived assuming all individuals had the same number of contacts. This allowed us to reduce the dynamics to three differential equations (after applying a moment closure approximation) tracking the changes in the number of pairs of the form [AB] . Now we relax the assumption of homogeneity, and track pairs for each class of individuals, where classes are defined by the total number of contacts an individual has. This analysis follows that presented in Eames and Keeling, 2002 [1].

Term	Description
a	rate of spontaneous infection
g	rate of recovery
β	rate of transmission through contact
$[n]$	# of individuals with n contacts
$[nm]$	# of pairs of individuals with n and m contacts
$[A]$	# of A individuals
$[A^n]^*$	# of A individuals with n contacts
$[A^nB^m]$	# of edges between an A^n and a B^m individual
$[A^nB]^* = \sum_m [A^nB^m]$	# of B contacts of all A^n's
$[A^nB^mC^q]$	# of triples with B^m having both A^n and C^q as contacts

Table 1: notation used in pairwise equations for heterogeneous networks

Table 1 summarizes the types of variables tracked with this approach. After describing some variables in terms of others, only those that are starred (*) remain, for a total of $3k$ equations, where k is the maximum number of contacts of any individual in the network. Whenever there is a sum, it is over all possible values for the number of contacts an individual has, i.e $\sum_n$ implies $\sum_{n=0}^{k}$. Note that while in the main text we wrote equations for the fraction of individuals in various classes, here we have left the equations for the absolute

numbers, for simplicity of notation.

$$
\begin{aligned}
&\frac{d}{dt}[I^n] = \beta[S^nI] + a[S^n] - g[I^n] \\
&\frac{d}{dt}[I^nI] = \sum_m \frac{d}{dt}[I^nI^m] = \beta(\sum_{m,q}([I^nS^mI^q] + [I^qS^nI^m]) + [S^nI] + [I^nS]) + a([S^nI] + [I^nS]) - 2g[I^nI] \\
&\frac{d}{dt}[S^nI] = \sum_m \frac{d}{dt}[S^nI^m] = \beta(\sum_{m,q}([S^nS^mI^q] - [I^qS^nI^m]) - [S^nI]) + a([S^nS] - [S^nI]) + g([I^nI] - [S^nI])
\end{aligned}
\tag{1}
$$

Many variables on the right hand side of these equations can be simplified until only $3k$ variables remain (equal to the number of equations). Firstly, triples can be reduced to pairs using the moment closure approximation [2]:

$$
[A^nB^mC^q] = \frac{(m-1)}{m}\frac{[A^nB^m][B^mC^p]}{[B^m]}(1 - \phi + \phi C_{A^nC^q}) \tag{2}
$$

$$
C_{A^nC^q} = \frac{[n][q]}{[nq]}\frac{[A^nC^q]}{[A^n][C^q]} \tag{3}
$$

$$
\tag{4}
$$

We still assume there is one ϕ that describes the whole population. We could have ϕ_{nmq}, though this would be unnecessarily complicated for most applications. Furthermore, we can approximate pairs of the type $[A^nB^m]$ in terms of the smaller set of pairs of the type $[A^nB]$ using:

$$
[A^nB^m] = \frac{[A^nB][B^mA]}{[AB]}\frac{[nm]\sum_q q[q]}{nm[n][m]} \tag{5}
$$

Finally, since all individuals are either infected or susceptible, we can use:

$$
\begin{aligned}
[I^nS] &= n[I^n] - [I^nI] \\
[S^nS] &= n[S^n] - [S^nI] \\
[I] &= \sum_n [I^n] \\
[S] &= N - [I]
\end{aligned}
\tag{6}
$$

This results in $3k$ equations and variables. If we want to find the spatial correlation discussed in the paper, we can use:

$$C_{AB} = \frac{[AB]}{\sum_{n,m} \frac{[nm]}{[n][m]} [A^n][B^m]} \tag{7}$$

References

[1] Eames, K. T. D. and Keeling, M. J. (2002). Modeling dynamic and network heterogeneities in the spread of sexually transmitted diseases. *Proceedings of the National Academy of Sciences of the United States of America*, 99(20):13330–13335.

[2] Rand, D. A. (1999). Correlation equations and pair approximations for spatial ecologies. In *Advanced ecological theory: principles and applications*, page 100. Wiley-Blackwell.
